# Amphipathic Liponecrosis Impairs Bacterial Clearance and Causes Infection During Sterile Inflammation

**DOI:** 10.1053/j.gastro.2023.05.034

**Published:** 2023-05-30

**Authors:** Sergiy Kostenko, Biswajit Khatua, Shubham Trivedi, Anoop Narayana Pillai, Bryce McFayden, Mahmoud Morsy, Prasad Rajalingamgari, Vijeta Sharma, Pawan Noel, Krutika Patel, Bara El-Kurdi, Henrique Borges da Silva, Xianfeng Chen, Vishal Chandan, Sarah Navina, Stacie Vela, Rodrigo Cartin-Ceba, Christine Snozek, Vijay P. Singh

**Affiliations:** 1 Department of Medicine, Mayo Clinic, Rochester, Minnesota; 2 Department of Immunology, Mayo Clinic, Rochester, Minnesota; 3 Department of Research Services, Mayo Clinic, Rochester, Minnesota; 4 Department of Pathology, School of Medicine, University of California, Irvine, California; 5 Clin-Path Associates, Tempe, Arizona; 6 Gastroenterology Section, Carl T. Hayden Veterans’ Administration Medical Center, Phoenix, Arizona; 7 Department of Pulmonary and Critical Care Medicine, Mayo Clinic, Scottsdale, Arizona; 8 Department of Laboratory Medicine and Pathology, Mayo Clinic, Scottsdale, Arizona; 9 Department of Biochemistry and Molecular Biology, Mayo Clinic, Scottsdale, Arizona

**Keywords:** VDAC, Infection, Fatty Acid, Immune Paralysis, Lipotoxicity

## Abstract

**BACKGROUND & AIMS:**

Although transient bacteremia is common during dental and endoscopic procedures, infections developing during sterile diseases like acute pancreatitis (AP) can have grave consequences. We examined how impaired bacterial clearance may cause this transition.

**METHODS:**

Blood samples from patients with AP, normal controls, and rodents with pancreatitis or those administered different nonesterified fatty acids (NEFAs) were analyzed for albumin-unbound NEFAs, microbiome, and inflammatory cell injury. Macrophage uptake of unbound NEFAs using a novel coumarin tracer were done and the downstream effects—NEFA–membrane phospholipid (phosphatidylcholine) interactions—were studied on isothermal titration calorimetry.

**RESULTS:**

Patients with infected AP had higher circulating unsaturated NEFAs; unbound NEFAs, including linoleic acid (LA) and oleic acid (OA); higher bacterial 16S DNA; mitochondrial DNA; altered *β*-diversity; enrichment in *Pseudomonadales*; and increased annexin V–positive myeloid (CD14) and CD3-positive T cells on admission. These, and increased circulating dead inflammatory cells, were also noted in rodents with unbound, unsaturated NEFAs. Isothermal titration calorimetry showed progressively stronger unbound LA interactions with aqueous media, phosphatidylcholine, cardiolipin, and albumin. Unbound NEFAs were taken into protein-free membranes, cells, and mitochondria, inducing voltage-dependent anion channel oligomerization, reducing ATP, and impairing phagocytosis. These were reversed by albumin. In vivo, unbound LA and OA increased bacterial loads and impaired phagocytosis, causing infection. LA and OA were more potent for these amphipathic interactions than the hydrophobic palmitic acid.

**CONCLUSIONS:**

Release of stored LA and OA can increase their circulating unbound levels and cause amphipathic liponecrosis of immune cells via uptake by membrane phospholipids. This impairs bacterial clearance and causes infection during sterile inflammation.

## Graphical Abstract

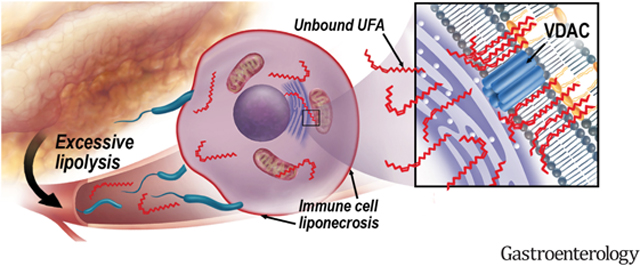

Transient bacteremia is common after toothbrushing, dental extractions,^1^ and endoscopic procedures.^2^ However diseases that start as sterile, such as acute pancreatitis (AP),^3^ trauma, and burns, can become infected and septic despite aseptic precautions. Such observations suggest impaired bacterial clearance converts sterile disease to infected disease; however, direct evidence for this and the underlying mechanisms are lacking. AP, which is acute inflammation of the viscerally located, normally sterile pancreas, provides an excellent opportunity to study these phenomena because AP can inexplicably result in infectious complications, including sepsis^3^ and local and systemic infections,^4,5^ without overt infectious exposure.

Fat necrosis, which is the rapid breakdown of triglyceride stored in visceral fat in and around the pancreas to nonesterified fatty acids (NEFAs), is common in AP.^6–10^ Fat necrosis results from leakage of pancreatic enzymes into the visceral fat during AP.^9–12^ NEFAs are generated by means of pancreatic triglyceride lipase entering the adipocyte via multiple mechanisms^13^ and hydrolyzing the triglyceride in lipid droplets to NEFAs.

Clues to the mechanisms of how AP may result in infection come from previous studies reporting lymphocyte apoptosis during AP^14^ and severe AP associated with a reduction in T-lymphocytes.^14,15^ Separately, lymphocyte apoptosis^16^ and active caspase-3 staining in the T-cell–rich splenic white pulp have been reported previously in clinical autopsies during non-AP sepsis.^17^ However, the mechanisms of immune cell injury, and whether they can convert a sterile disease to an infected one, are unknown.

Therefore, using rodent, cellular, and biochemical models, we studied whether, during AP, NEFAs released from visceral triglyceride lipolysis can injure immune cells and impair their ability to clear organisms. We compared emergency department samples from patients with infected AP (ie, those who developed infections or sepsis, those with microorganisms isolated, or those requiring antibiotics for clinical suspicion of infection) with those from patients with noninfected AP and healthy controls and conducted autopsy studies. Lipidomic and microbiome parameters were studied, along with how hydrophobic NEFAs in our aqueous environments may injure immune cells when unbound to their carrier albumin,^18,19^ which is reduced early in severe AP.^20,21^ We noted amphipathic, unbound, unsaturated NEFA–phospholipid interactions increase their cellular uptake, causing immune cell mitochondrial injury, inflammatory cell death, and impaired bacterial clearance, thus causing infection during a sterile disease.

## Methods

### Human Studies

All human data were collected in compliance with consent, access to medical records, and sample collection criteria approved by the Institutional Review Boards at the Mayo Clinic Foundation and Carl T. Hayden Veterans Affairs Medical Centers, as described previously.^22^ Patients presenting to the Mayo Clinic Arizona emergency department between September 2019 and July 2021 who fulfilled two-thirds or more of the AP diagnostic criteria as per the Revised Atlanta Criteria guidelines^23^ were considered for inclusion initially. Controls were healthy patients without pancreatitis and presenting for elective outpatient visits or were actively recruited for the study. The serum handling is detailed under microbiome studies in the Supplementary Material. Flow cytometry was performed in patients who had a citrate or heparin blood vial available. In the context of our studies, citrated blood samples are sometimes collected for patients with an unclear cause of abdominal pain, as per the judgment of the emergency physician, as are heparinized samples, which are collected for point-of-care tests. These samples were preferred over EDTA samples because citrate (used as an anticoagulant for blood transfusion) and heparin (used as an anticoagulant for extracorporeal circuits like hemodialysis) are less toxic. Medical records of the patients with AP were reviewed for a dismissal diagnosis of sepsis, positive cultures or stains for microorganisms, clinical documentation of a suspicion for infection resulting in administration of antibiotics, and biochemical parameters at the time of admission. Patients with active malignancy, immunosuppression (eg, biologic agent), chemotherapy, and aged older than 90 years were excluded from the study. Normal controls included all of the healthy nonhospitalized individuals who were age-, sex-, and body mass index–matched to the patients with AP, and had samples analyzed or available for the relevant lipidomic and microbiome analysis. At the time of assays, the individuals or institutions performing them (lipidomics at Vanderbilt University and microbiome at Arizona State University) were blinded to the identity of the samples.

The remaining Methods are detailed in the Supplementary Material.

## Results

### An Increase in Serum Nonesterified Fatty Acids and Their Unbound Forms Is Associated With Significant Infection During Clinical Acute Pancreatitis

**Figure 1.**
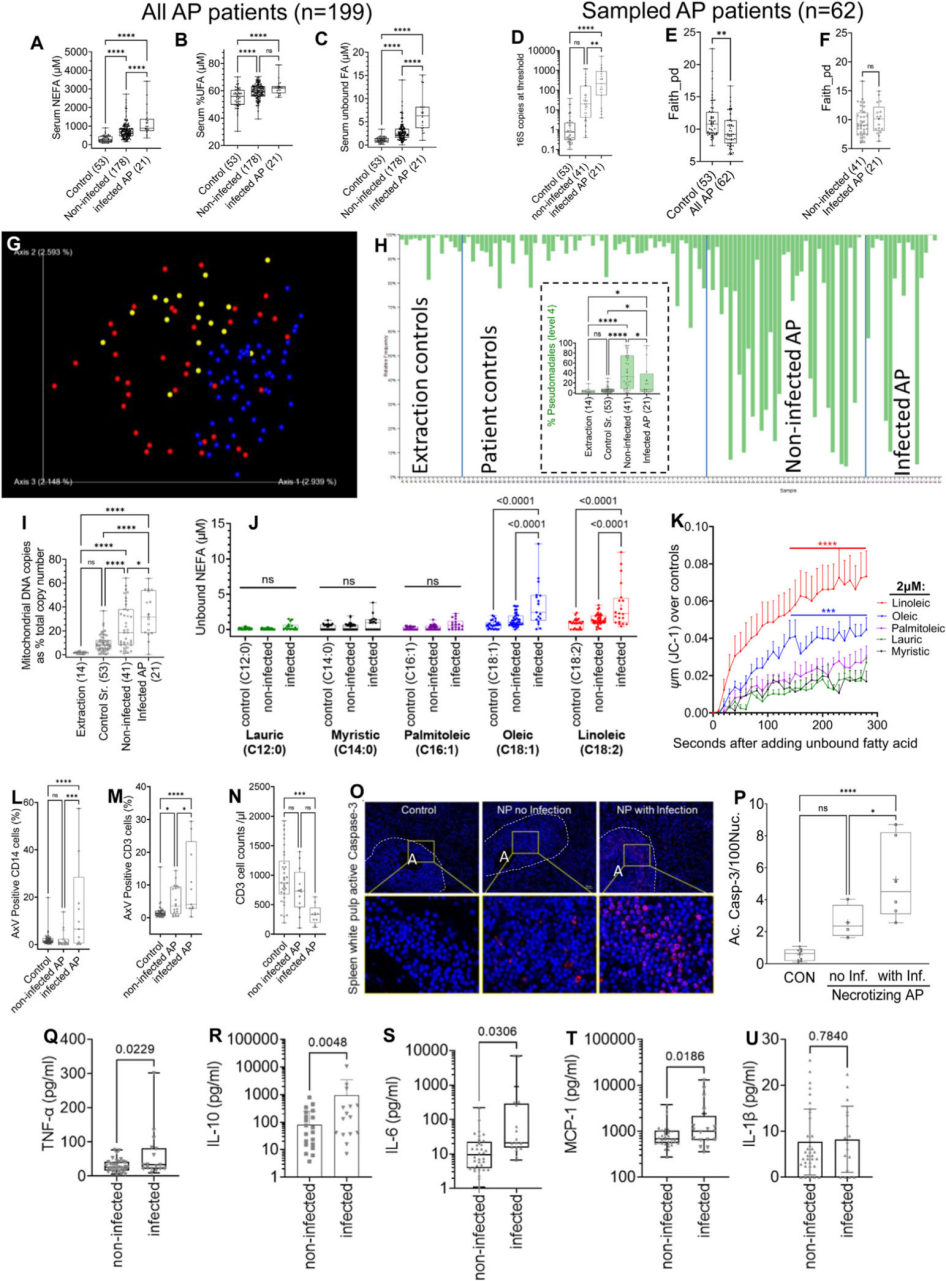
Findings in clinical AP cases and controls. *Boxplots* showing admission serum NEFAs (*A*), percent unsaturated FAs of total NEFAs (*B*), fluorimetrically determined unbound NEFAs (*C*), 16S DNA copy number (*D*), *faith plots* of microbial *α*-diversity in controls and patients with AP (*E*), and patients with infected and noninfected AP (*F*). (*G*) Jaccard-Emperor (*β*-diversity) *plot* showing controls (*blue*), noninfected (*red*), and infected (*yellow*) patients. (*H*) Level 4 taxa *bar plots* of percent *Pseudomonadales* in different groups, summarized in *inset boxplot*. *Boxplots* comparing percent mitochondrial DNA (*I*) and micromolars of unbound NEFA (*J*) in patient samples. (*K*) Time course of mitochondrial depolarization (ψm) in peritoneal macrophages (analysis of variance) induced by 2 *μ*M unbound NEFA in *colors* mentioned on *right*. Flow cytometry data comparing percent annexin V–positive (AxV) CD14 (*L*), CD3 (*M*) cells, and CD3 cells (counts/*μ*L) (*N*). Spleen white pulp cells (*blue nuclei* in *dashed white outline* surrounding the central arteriole A) staining for active caspase-3 (*red*) on autopsies of controls, patients with necrotizing pancreatitis (NP) with no infection and NP with infection (*O*). (*P*) Comparison of percent positive cells in the 3 groups. (*Q*–*U*) Serum cytokines (y-axis) in the admission sera of patients with AP.

Among 199 patients with AP, 21 (excluding those for endoscopic retrograde cholangiopancreatography and cholecystectomy) required antibiotics for suspected infection. Thirteen had microorganisms isolated and 13 were diagnosed with sepsis. Compared with age-, sex-, and body mass index–matched normal controls (n = 53), patients with AP had higher serum lipase (Table 1). As seen in Figure 1A–C, on analysis of variance, infected patients had higher serum NEFAs (1196 ± 784 *μ*m, 749 ± 427 *μ*M, and 297 ± 155 *μM*, respectively), and fluorimetrically measured unbound fatty acids (FAs) (6.9 ± 4.1 *μ*M, 2.8 ± 1.9 *μ*M, and 1.0 ± 0.6 *μM*, respectively), and longer length of stay (18.3 ± 18.4 days vs 3.4 ± 3.0 days; *P* < .0001) than noninfected patients (n = 178) or controls. For more detailed, intensive (microbiome and lipidomics) studies, we chose 41 age-, sex-, body mass index–, and AP etiology–matched patients with noninfected AP to compare with the 21 infected patients (Table 2). These 41 had serum NEFAs and unbound NEFAs (Table 2) similar to those of the 178 noninfected controls (Figure 1A–C). Table 2 shows the site of infection and a lower serum albumin in infected vs noninfected controls. Overall, these 62 patient who underwent more detailed studies (Table 1) had similar demographic characteristics, AP etiology, serum lipase, and basic lipid parameters compared with the total 199 patients with AP.

### Infected Acute Pancreatitis Is Associated With Increased Circulating Bacterial 16S DNA and Unbound, Long-Chain, Unsaturated Fatty Acids That Can Cause Immune Cell Injury

To determine how unbound NEFAs may increase the risk of infection in a sterile disease, we studied 16S bacterial DNA amounts, microbial diversity, type of unbound NEFAs, and immune cell injury by means of flow cytometry in the 21 infected, 41 noninfected patients, and 53 controls. Each patient had at least 1 sample available for each of these assays.

We first compared serum 16S bacterial DNA amounts at the threshold cycle number of extraction controls (26 cycles). The sera of patients with infected AP had significantly higher 16S bacterial DNA copies (669 ± 1209/10 *μ*L) than noninfected patients (170 ± 340/10 *μ*L), or normal controls (2.8 ± 6.3/10 *μ*L) (Figure 1D). The cell-free DNA was then amplified to 35 cycles for microbiome analysis. Samples with >10,000 copies (patients with AP averaged >120,000 copies) were analyzed (Supplementary Figure 1). After exclusion of unidentified DNA (see below), patients with AP had significantly lower phylogenetic *α*-diversity (*P* = .00138; Figure 1E) than controls. This was irrespective of infection (Figure 1F). *β*-diversity was altered during AP (*red* and *yellow dots* in Figure 1G) vs controls (*blue dots* in Figure 1G), as seen the Jaccard-Emperor plot (*P* < .001; permutational multivariate analysis of variance). Taxonomic abundance comparison at level 4 (order) showed patients with AP had an increased proportion of *Pseudomonadales* (Figure 1H). These included *Acinetobacter* and *Pseudomonas*, which are common human pathogens in AP^4,5^ (linear discriminant analysis in Supplementary Figure 2). Three among 6 blood cultures collected at the same time were positive for bacterial growth in the infected group. These 3 had a high proportion of the cultured organism on microbiome analysis (percentages in parentheses), including *Escherichia coli* (21.7%), *Clostridium perfringens* (81%), *Cronobacter* (14%), and *Staphylococcus aureus* (12%), thus supporting the clinical relevance of the microbiome data. Interestingly, the sequences amplified with the 16S primers also contained unidentified sequences (*gray bars* in Supplementary Figure 3), which, on analysis against the human genome, were enriched in mitochondrial DNA. There was no significant difference in total number of DNA copies amplified at 35 cycles among the groups (Supplementary Figure 3), and mitochondrial DNA comprised <2% of the total amplified sequences in the extraction controls. In contrast, the proportion of mitochondrial DNA significantly increased in patients with infected AP to 34% ± 18% (Figure 1I) compared with normal human sera (9.7% ± 7.1%) or noninfected AP cases (23.6% ± 18.8%), suggesting increased mitochondrial injury early during infected AP.

Because unbound NEFAs are higher in infected patients (Figure 1C) and can injure mammalian mitochondria,^24,25^ we de-albuminated sera to measure individual unbound NEFAs by means of gas chromatography mass spectrometry. These were measured in the sera of 21 patients with infected AP, 41 patients with noninfected AP, and one-half (n = 27) of the randomly selected controls (Figure 1J). Unbound lauric, myristic, and palmitoleic acid remained unchanged in all groups. Infected patients had higher unbound oleic acid (OA) (3.5 ± 3.1 *μ*M) and linoleic acid (LA) (3.4 ± 3.1 *μ*M), which remained unchanged in noninfected patients (1.3 ± 0.9 *μ*M vs 0.7 ± 0.6 *μ*M) for OA, or LA (1.4 ± 0.8 *μ*M vs 0.8 ± 0.7 *μ*M) vs controls (Figure 1J). Although levels of unbound stearic acid (C18:0) and palmitic acid (PA) (C16:0) did not change among groups, background interference prevented their accurate measurement in the low unbound range and were thus not included in the analysis.

To identify which unbound NEFAs injure inflammatory cells and may impair bacterial clearance in infected patients, JC-1–loaded mouse peritoneal macrophages in a stirred cuvette were exposed to 2 *μ*M (ie, > 75^th^ percentile in noninfected patients) of different unbound NEFAs. Unbound OA and LA significantly increased mitochondrial depolarization (ψm) by 2–4 times over other NEFAs (Figure 1K).

Therefore, infected AP increases serum unbound OA and LA (3–4 *μ*M vs <1 *μ*M in controls),^25^ which induce ψm in macrophages. We therefore looked for evidence of inflammatory cell injury in the patients with AP.

### Patients With Infected Acute Pancreatitis Injure and Reduce the Immune Cells in Their Circulation

Because unbound NEFAs in infected sera may cause immune cell injury (Figure 1K), which may impair bacterial clearance, we went on to study whether immune cell death was increased in infected AP using flow cytometry. Compared with controls, patients with infected AP had a higher percentage of annexin V–positive CD14^+^ myeloid cells (15.0% ± 20% vs 2.2% ± 3.0%; *P* < .0001) and CD3 cells (10.9% ± 11.3% vs 1.7% ± 2.2%; *P* < .0001) (Figure 1L and M and Supplementary Figure 4). This was significant enough to reduce the circulating CD3^+^ T cell number from 966 ± 460/*μ*L in controls to 329 ± 169/*μ*L in patients with infected AP (*P* < .001; Figure 1N). We confirmed the relevance of T-lymphocyte injury on autopsies of patients who had died from infected pancreatic necrosis vs matched controls who died from unrelated causes (Supplementary Figure 5). On examining the T-cell–rich^17^ splenic white pulp (surrounding the central arteriole A in Figure 1O) for active caspase-3 by immunohistochemistry (Figure 1O and P), we noted this increased from 0.6% ± 0.4% in controls to 5.2% ± 2.5% in patients with infected necrosis (*P* < .0001). Infected patients’ sera had higher cytokines (Figure 1Q–U), which in rodents results from unbound NEFAs (shown later). Thus, immune cell injury in patients with infected AP may result from elevated circulating unsaturated FAs that are unbound to albumin. We therefore went on to study the underlying mechanisms.

### Unbound Fatty Acid Uptake Via Membrane Phospholipid Interaction Causes Mitochondrial Depolarization and Voltage-Dependent Anion Channel Oligomerization, Reduces ATP, and Impairs Phagocytosis

**Figure 2.**
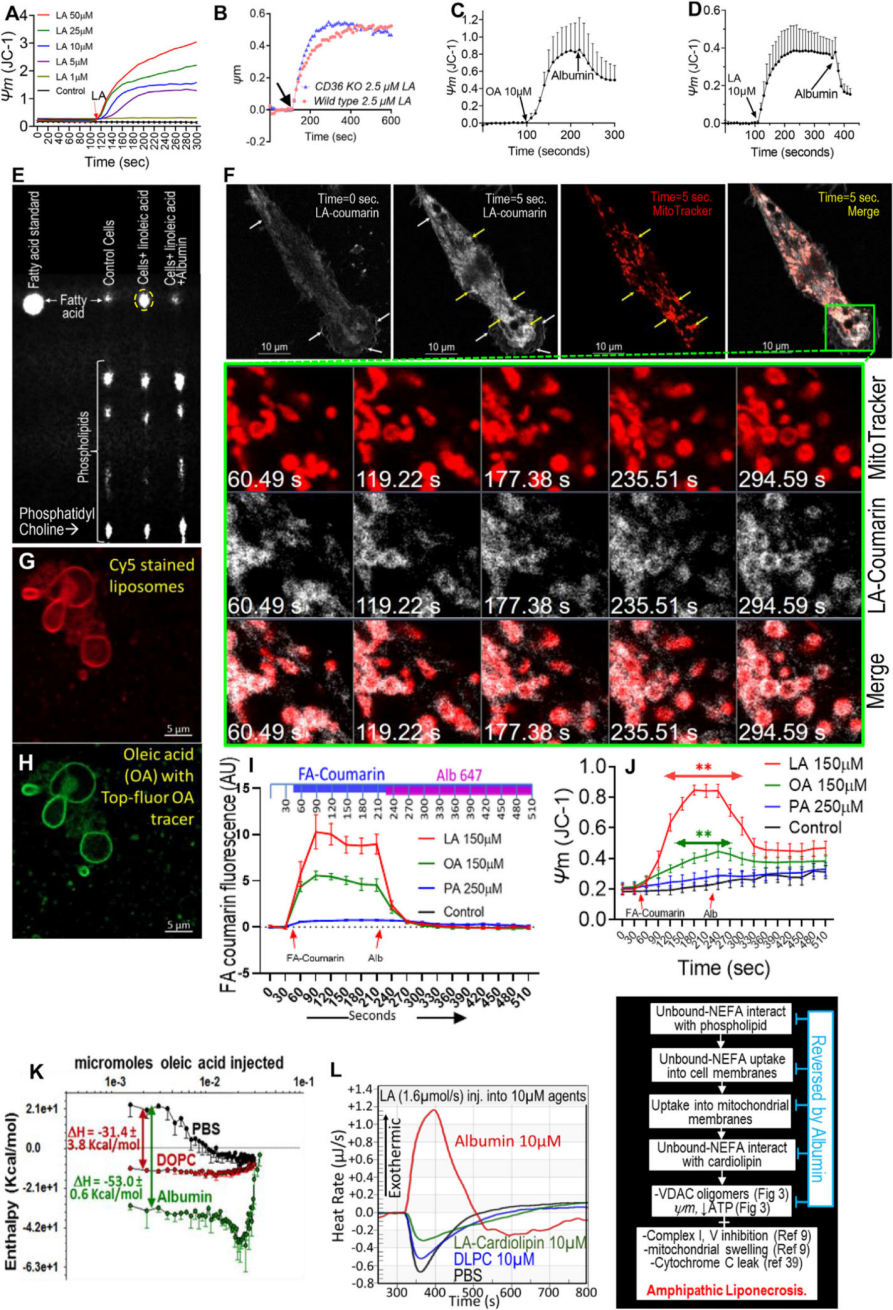
Unbound NEFA uptake in macrophages and resulting mitochondrial depolarization (ψm), in relation to strength of interactions with phospholipids and albumin on ITC: Time course of ψm induced by unbound LA in J774A.1 cells at different doses (*A*), at 2.5 *μ*M in in peritoneal macrophages from wild-type (*pink*) or CD36 knockout (*blue*) mice. Reversal of 10 *μ*m OA (*C*) and LA (*D*) induced ψm by 0.1% albumin added later. (*E*) Thin-layer chromatography *image* of lipid extracts from J774A.1 control cells (*left lane*), after 2 minutes of 50 *μ*m LA (*middle lane*) and addition of 0.5% albumin after 2-minute LA (*right lane*). The FA standard and location is shown in the *left-most lane*. (*F*) Live imaging (Supplementary Video 1) of LA, LA-coumarin uptake (*white*) on addition (0 second, *left-most*), 5 seconds later (*second from left*), along with MitoTracker red (*red*), and *combined images*. *White arrows* show cell membrane, and *yellow arrows* mitochondrial staining. *Green inset*, *zoom* show *timed images*. *Images* of Cy5 stained liposomes (*G*) after exposure to OA with Top-fluor tracer (*H*). *Images* of Cy5-stained liposomes (*G*) after exposure to OA with Top-fluor tracer (*H*). (*I*) Time courses (Supplementary Videos 2–4) comparing uptake of different coumarin FAs (mentioned on *right*) by J774A.1 cells and their extraction by albumin (Alb) added at 210 seconds. (*J*) ψm (using JC-1) in J774A.1 cells shown in Figure 2G and their reversal by Alb added. (*K*) *Enthalpograms* (mean with SEM) of intermittent injections of OA into PBS (*black*), dioleyl phosphatidylcholine (DOPC; *red*), and Alb (*green*). The ∆H depicts the enthalpy change compared with PBS. (*L*) Raw heat rate average of continuous injection of LA (1.6 *μ*m/s) into PBS vs 10 *μ*m of dilinolyl-phosphatidyl choline (DLPC), or LA cardiolipin, or Alb. (*M*) Event sequence from unbound NEFA uptake until amphipathic liponecrosis.

To understand the mechanisms underlying unbound NEFA–mediated immune cell injury in vivo, we exposed JC-1–loaded J774A.1 macrophages in a stirred cuvette to increasing concentrations of unbound LA and noted a dose-dependent increase in ψm (Figure 2A). This ψm was not reduced in peritoneal macrophages from CD36 knockout mice (Figure 2B) or FFAR4/GPR120 knockout mice (data not shown). Addition of the FA carrier albumin 2–5 minutes after unbound OA (Figure 2C) or LA (Figure 2D), however, rapidly reversed the ψm, suggesting unbound FA-mediated ψm is non-receptor–, non-transporter–dependent.

We then studied whether unbound NEFAs could be reversibly taken up into cells. Thin-layer chromatography of control J774A.1 cell lipid extracts showed these to be mainly phospholipids (Figure 2E, *first lane*), including phosphatidyl choline (*bottom of plate*). LA, when added to cells in albumin-free medium, was taken up into the cells (Figure 2E, *yellow oval, middle lane*). This uptake was reversed by adding albumin 2 minutes later (Figure 2E, *last lane*). We then visualized FA uptake using LA with a tracer fluorophore (3-hydroxycoumarin) attached to the methyl terminus (LA-coumarin at 2% of total LA concentration). LA-coumarin was reproducibly visualized in cells at approximately 1 *μ*M (total LA > 30 *μ*M; Supplementary Figure 6) as detailed in the Methods. LA-coumarin initially localized to the plasma membrane of J774A.1 cells (Figure 2F, time = 0 seconds, *white arrows*). Most LA then rapidly enriched around MitoTracker–positive mitochondria (Figure 2F, *yellow arrows*, time = 5 seconds, Supplementary Video 1). The congruence of LA-coumarin with the moving mitochondria is apparent in the timed *images* within the *zoomed green rectangle*. We then studied whether unbound OA partitions into protein-free phospholipid membranes using palmitoyl-oleyl phosphatidyl choline liposomes with Cy5 tracer (Figure 2G). OA (with Topfluor tracer; *green*), when added to the medium, partitioned into liposome membranes (Figure 2H), supporting a protein-independent uptake. In J774A.1 cells, uptake of LA > OA > PA (Figure 2I and Supplementary Videos 2–4), despite PA’s higher concentrations (250 *μ*M) and solvent (0.3% dimethyl sulfoxide; ie, 42 *μ*M) requirement. This parallels the ability of LA, OA, and PA to be unbound monomers at approximately 160 *μ*M, 40 *μ*M, and <8 *μ*M, respectively, in aqueous media at 37°C.^25^ The uptake of LA and OA significantly depolarized mitochondria (Figure 2J), which was also reversed by albumin.

We then compared the energetics of unbound LA and OA interactions with phosphatidylcholine using isothermal titration calorimetry (ITC; Figure 2K) at 37°C in phosphate-buffered saline (PBS). ITC compared interactions of unbound NEFA with PBS in which they are present vs the membrane phospholipids they partition into (Figure 2E–H) vs albumin, which reverses their cellular uptake and responses (Figure 2C–E, I–J). On consecutive injection ITC, OA interacted most favorably with albumin, followed by the phospholipid dioleyl phosphatidyl choline (Figure 2K, *red line*) and then PBS (*black line*); potentially explaining why OA may partition into phospholipid-rich membranes, which is reversed by albumin (Figure 2E and I). Quantitatively, the interaction with albumin was more favorable (shown as −ΔH of −53 kcal/mol) vs dioleyl phosphatidyl choline (−31.4 ± 3.8 kcal/mol) vs PBS. Similarly, LA interacted more favorably with its phospholipid dilinolyl phosphatidyl choline than with PBS (data not shown). This was verified on continuous injection (Figure 2L), wherein LA–dilinolyl phosphatidyl choline interactions (*blue line*) were more exothermic than LA–PBS interactions (*black line*). Interestingly, LA cardiolipin; a phospholipid at inner–outer mitochondrial membranes, and endoplasmic reticulum–mitochondria contact points^26–28^ with 4 LA chains interacted with LA more strongly (Figure 2J, *green line*) than dilinolyl phosphatidyl choline (*blue line*), explaining the enrichment of LA-coumarin around mitochondria (Figure 2F). This sequence of events triggered by unbound NEFA uptake are summarized in Figure 2M.

**Figure 3.**
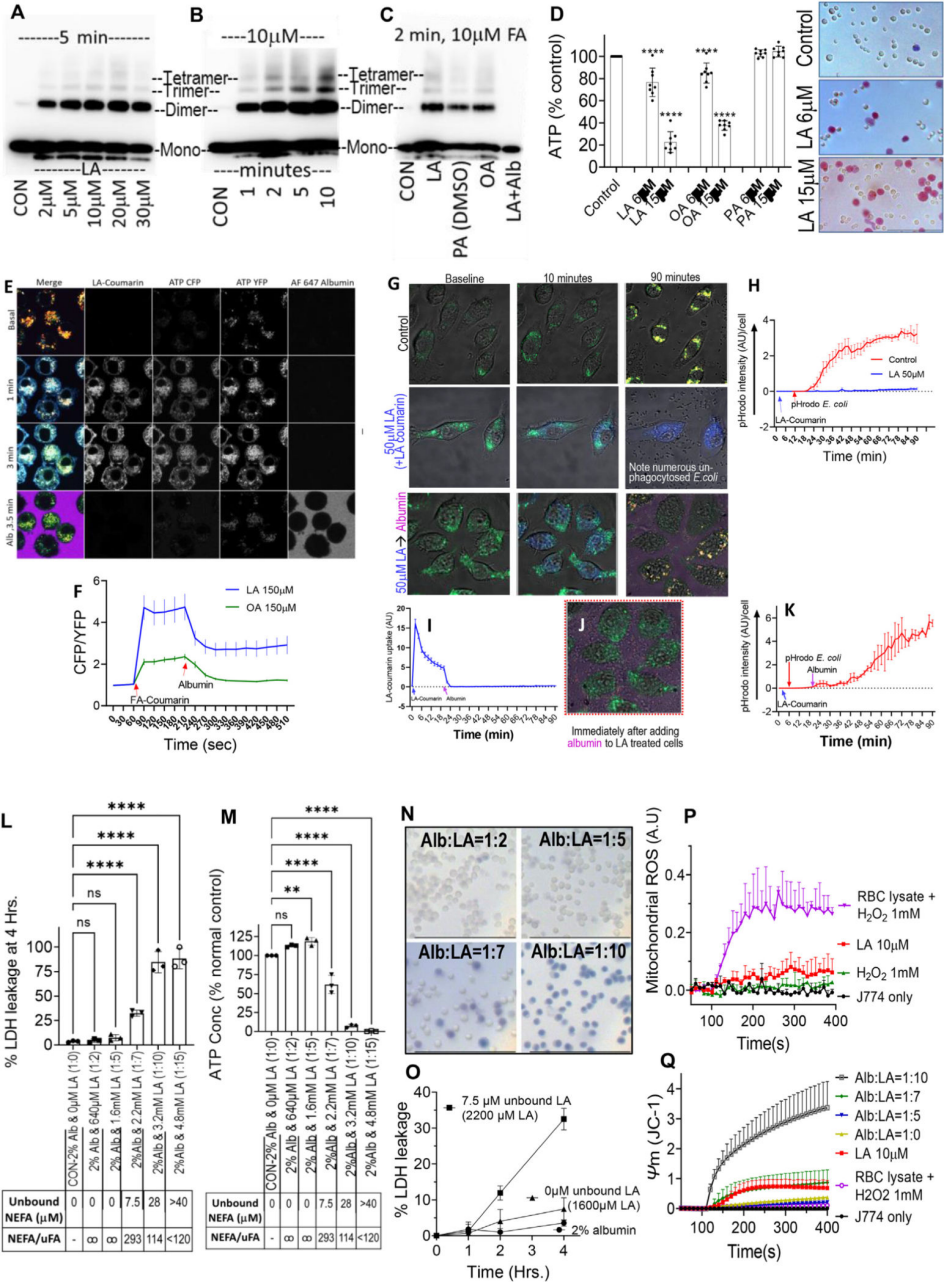
Unbound FA–induced mitochondrial and phagocytic changes in J774A.1 cells: *Western blots* showing VDAC oligomerization induced by different concentrations (*A*), and different times after adding 10 *μ*M unbound LA (*B*). (*C*) Extent of VDAC oligomerization induced by 10 *μ*M unbound NEFA at 2 minutes. Note, LA and OA had no solvent, and PA required 0.3% (42 mM) dimethyl sulfoxide (DMSO). The *last lane* shows the effect of albumin (0.2%) added 2 minutes after LA. (*D*) ATP concentrations, trypan blue staining (*right-side images*) after 1 hour of unbound NEFA exposure in peritoneal macrophages. (*E*) Fluorescence resonance energy transfer–based ATP levels in ATeam sensor–transfected cells (Supplementary Videos 5 and 6) showing baseline YFP fluorescence (*top row*) and increase in cyan fluorescence after 1 and 3 minutes (*middle rows*) of LA-coumarin. Note loss of CFP and LA-coumarin fluorescence with albumin (with Alexa 647 tracer; *bottom row*). (*F*) *Graph* of ATP reduction (CFP/YFP ratio) after adding FA at 60 seconds, and its reversal by adding albumin later. (*G*) 90-minute confocal time series of pHrodo *E coli* phagocytosis (Supplementary Videos 7–9) by Lysotracker green–loaded J774A.1 cells. Controls are *top row*, LA with coumarin tracer are *middle row*, and LA-coumarin followed by albumin (with tracer) addition at 20 minutes are *bottom row*. (*H*) Quantifies pHrodo *E coli* uptake in control (*red tracing*) and LA-treated (*blue*) cells. *Graphical depiction* of reversal of LA-coumarin uptake (*I*) and restoration of pHrodo *E coli* phagocytosis (*K*) after adding albumin. (*J*) *Image* of cells just after the addition of albumin at 20 minutes: note loss of LA-coumarin seen at 10 minutes. *Bar graphs* showing effect of different albumin to LA conjugate ratios on lactate dehydrogenase (LDH) leakage (*L*), ATP concentrations normalized to controls (*M*). Unbound LA concentrations are shown in the *boxes below*. (*N*) Trypan blue staining after 1 hour of different albumin to LA ratios. (*O*) Time course of LDH leakage, MitoSox fluorescence (*P*), mitochondrial depolarization (*Q*) under conditions mentioned.

We then examined the impact of unbound NEFA uptake on oligomerization of the voltage-dependent anion channel (VDAC), monomers of which are normally separated by cardiolipin.^29^ The cellular uptake of LA and OA caused oligomerization of VDAC in a dose- (Figure 3A) and time-dependent manner (Figure 3B). The oligomers formed again were LA > OA > PA, and were reversible by albumin (Figure 3C), consistent with the relative strength of FA–phospholipid vs FA–albumin interactions we noted on ITC (Figure 2I–J), and unbound monomeric FA concentrations these FAs can achieve in aqueous environments.^25^

Because VDAC transports ATP^30^ from mitochondria and its oligomerization can trigger apoptosis,^31^ we measured ATP by luminescence (Figure 3D) in peritoneal macrophages after 1-hour treatment. Unbound LA and OA dose-dependently reduced ATP levels and increased trypan blue staining in necrotic cells, unlike PA, which is more hydrophobic and not able to exist as monomers when unbound to albumin^25^ (Figure 3D). We then verified that this was due to unbound NEFA by means of real-time ATP measurements using the ATeam 1.03 Fluorescence resonance energy transfer sensor^32^ for live imaging of ATP levels (Figure 3E and F and Supplementary Videos 5 and 6). At baseline, J774A.1 cells had a low CFP/YFP ratio, with the ATP-induced YFP fluorescence localizing to a perinuclear mitochondria-like morphology (Figure 3E, *top row*), as reported previously.^32^ Uptake of LA or OA at concentrations that clearly depolarized mitochondria (150 *μ*M; Figure 2G, Supplementary Figure 6C) increased CFP fluorescence consistent with reduced ATP levels (Figure 3E and F, *middle row*). These changes were rapidly reversed by exogenous albumin (Figure 3E, *bottom row*). To determine whether this reduction in ATP impacted cell function, we then studied the impact of unbound LA on the ability J774A.1 cells to phagocytose pHrodo *E coli*. The normal phagocytosis of pHrodo *E coli* by J774A.1 cells (Figure 3G, *top row*; Figure 3H, *redline*) was completely prevented by unbound LA (Figure 3G, *middle row*; Figure 3H, *blue line*; and Supplementary Videos 7 and 8). Addition of exogenous albumin 20 minutes after addition of the LA partially rescued this impairment in phagocytosis (Figure 3G, *third row*, Figure 3I–K, Supplementary Video 9), while reversing the uptake of unbound LA.

We further confirmed the role of unbound NEFA in J774A.1 injury by conjugating albumin (2%; 320 *μ*M final concentration) to LA at ratios from 1:0 to 1:15, and measuring unbound LA, mitochondrial depolarization, ATP, and cell injury (Figure 3L–Q). Unbound LA was undetectable at ≤1:5 ratio (total LA ≤1.6 mM; *table below* Figure 2L and M). Unbound LA increased from 7.5 *μ*M at 1:7 (total LA 2.2 mM) onwards as shown. Lactate dehydrogenase leakage at 4 hours increased with unbound LA, and ATP levels at 1 hour decreased (Figure 2L and M). Trypan blue uptake paralleled the ATP drop, with lactate dehydrogenase leak increasing over time; consistent with necrosis (Figure 3N–O). MitoSox-loaded cells were used to study mitochondrial reactive oxygen species (MROS) generation. Although LA did not affect MROS generation (AU; Figure 3P, *red line*), addition of hydrogen peroxide (1 *μ*M) with hemoglobin (50 *μ*g/dL, which has a peroxidase activity) increased MROS significantly (Figure 3P, *purple line*). However, this did not depolarize mitochondria, unlike 7.5–28 *μ*M unbound LA (Figure 3Q, *red*, *green*, *black lines*). Notably 7.5 *μ*M unbound LA (from 2.2 mM total LA conjugated to 2% albumin, *green line*) was similar to 10 *μ*M unbound LA without albumin (*red line*).

Overall, these studies indicated that interaction of unbound NEFA with membrane phospholipids results in their uptake into cells, with consequent mitochondrial depolarization (independent of MROS), VDAC oligomerization, reduced ATP levels, impairment of phagocytosis, and progression to necrosis. The reversibility of early phenomena by albumin (Figure 3I and K) is consistent with the strong binding of FAs to albumin (Figure 2K and L) These findings prompted us to evaluate the relevance of this to impairment of bacterial clearance in vivo.

### Unbound Nonesterified Fatty Acids Cause Immune Cell Injury and Increase Bacterial Loads in vivo, Causing Infection During Sterile Inflammation

**Figure 4.**
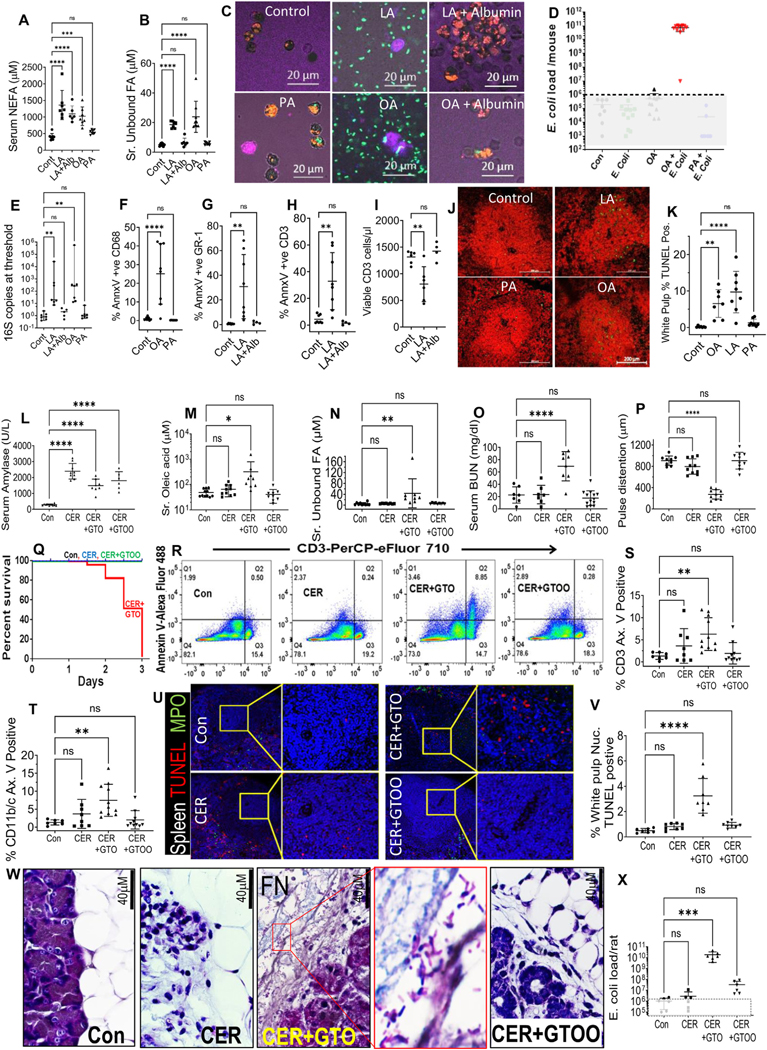
Effect of FA type, unbound levels on bacterial clearance, and immune cell injury in vivo: Serum levels of NEFAs (*A*) and unbound NEFAs (*B*) in mice given the mentioned NEFAs alone or after preconjugation with albumin. (*C*) *Images* showing effects of intraperitoneal NEFAs ± albumin preconjugation on clearance of 10^6^ GFP *E coli* (*green*), phagocytosis of *pHrodo E coli* (*red*), and live dead marker staining (*purple*). (*D*) GFP *E coli* load/mouse at the end of experiment shown in (*C*). (*E*–*K*) are from mice not given *E coli.* (*E*) Bacterial 16S DNA copy number, flow cytometry showing percentage of annexin V–positive CD68^+^ (*F*), GR-1^+^ (*G*), CD3^+^ (*H*) cells, and number of viable CD3^+^ cells/*μ*L (*I*). (*J*) Spleen white pulp terminal deoxynucleotidyl transferase–mediated deoxyuridine triphosphate nick-end labeling (TUNEL) staining *images* (*green* indicates positive cells). (*K*) Quantification of (*J*). (*L*) Serum amylase *scatterplots* after 24 hours, rat CER pancreatitis with triolein (GTO) or triolein+orlistat (GTOO) compared with controls (Con). (*M*–*X*) shows 3-day parameters of serum OA (*M*), unbound NEFAs (*N*), blood urea nitrogen (*O*), and shock measured by carotid pulse distention (*P*). (*Q*) *Survival curve*. (*R*) *Scattergrams* of annexin V, CD3^+^ staining in blood. *Scatterplots* of CD3^+^ (*S*), %CD11b/c^+^, and (*T*) annexin V^+^ cells. (*U*) TUNEL (*red*), myeloperoxidase (MPO) staining (*green*) of spleen (nuclei, *blue*) highlighting white pulp (*inset*). (*V*) *Scatterplots* comparing %TUNEL^+^ white pulp cells. (*W*) Gram-stained images (100×) of pancreas–fat interface of each condition showing mixed flora bacteria (*zoomed inset*) in CER+GTO fat necrosis (FN). (*X*) GFP-*E coli* numbers/rat peritoneal cavity, *gray boxes* in (*D*, *X*) show lower limit of calibration curve.

To study the relevance of unbound NEFAs on bacterial clearance in vivo, we first administered LA, OA, or PA to mice intraperitoneally and measured serum levels. LA was administered in 2 forms, that is, LA alone or after prebinding to albumin (LA + Alb), as described previously.^24^ LA in both forms and OA increased serum NEFAs, and PA, perhaps due its extreme hydrophobicity, did not increase serum NEFAs (Figure 4A). Prebinding of LA to albumin prevented the increase in serum unbound NEFA levels, unlike LA and OA alone (Figure 4B). Because albumin binding prevented unbound FA increase, and in vitro studies in Figure 3 showed albumin to rescue LA-induced impairment in phagocytosis, we next compared whether prebound LA or OA protected from immune cell injury and impairment of bacterial clearance induced by LA or OA in vivo. For this, as described in the Methods, mice were given GFP *E coli* intraperitoneally 2 hours after the FAs, and pHrodo *E coli* 2 hours before necropsy, followed by live dead marker staining of the peritoneal fluid. In control and PA-treated mice (Figure 4C), pHrodo *E coli* were noted to be phagocytosed, and most cells were viable with some of the cells containing GFP *E coli*, and no bacteria in the extracellular fluid. Mice given LA and OA had large increases in GFP *E coli* in the peritoneal cavity (approximately 10^11^/mouse, Figure 4D), with the peritoneal cells appearing necrosed (Figure 4C, *middle panels*), with no evidence of phagocytosis of the pHrodo *E coli*. However, in mice where the OA and LA had been prebound to albumin, the pHrodo *E coli* was phagocytosed into viable peritoneal macrophages, with no visible GFP *E coli* in the extracellular fluid (Figure 4C, *right panels*). Similar to our infected patients (Figure 1D), LA and OA increased 16S DNA copies in the serum, unlike LA conjugated to albumin in mice given PA (Figure 4E). Like in infected patients (Figure 1L), OA caused myeloid (CD68^+^) cell injury unlike PA (Figure 4F). Similarly, within 4–5 hours LA alone (unlike albumin-bound LA) increased annexin positivity in GR-1^+^ myeloid and CD3^+^ T cells (Figure 4G and H, Supplementary Figures 8 and 9), and reduced viable CD3^+^ T cell counts in the circulation (Figure 4I). Unlike PA, both LA and OA increased terminal deoxynucleotidyl transferase–mediated deoxyuridine triphosphate nick-end labeling–positive apoptotic cells in the splenic white pulp (Figure 4J and K), similar to patients dying from infected necrosis (Figure 1O and P). These studies found that albumin-unbound NEFAs in vivo may cause immune cell injury and increase bacterial loads in vivo. We, therefore, went on to study whether unbound NEFAs generated during caerulein (CER)-^10^ or interleukin (IL)12,18-^9,25^ induced AP ± visceral triglyceride lipolysis could mimic the phenotype of increased bacterial loads in our patients with infected AP (Figure 1). CER AP increased serum amylase in all groups (Figure 4L). Lipolysis of the OA triglyceride (glyceryl trioleate [GTO]) increased serum OA, unbound NEFAs in the CER+GTO group, which was prevented by the lipase inhibitor orlistat (CER+GTOO; Figure 4M and N). CER+GTO induced renal failure with blood urea nitrogen elevation, as reported previously,^10^ shock (noted as pulse distention), and reduced survival (Figure 4O–Q and Supplementary Figure 10). CER+GTO increased annexin V staining in CD11b/c^+^, CD3^+^ cells, and terminal deoxynucleotidyl transferase–mediated deoxyuridine triphosphate nick-end labeling–positive cells in the splenic white pulp (Figure 4R–V). All of these were prevented in the CER+GTOO group. Gram staining showed a mixed flora extending into the damaged pancreas from fat necrosis in the GTO+CER group (Figure 4W, *zoomed inset*). Separately, in rats given 10^7^ GFP *E coli* via gavage, only the CER+GTO had higher peritoneal bacterial loads unlike other groups (Figure 4X).

**Figure 5.**
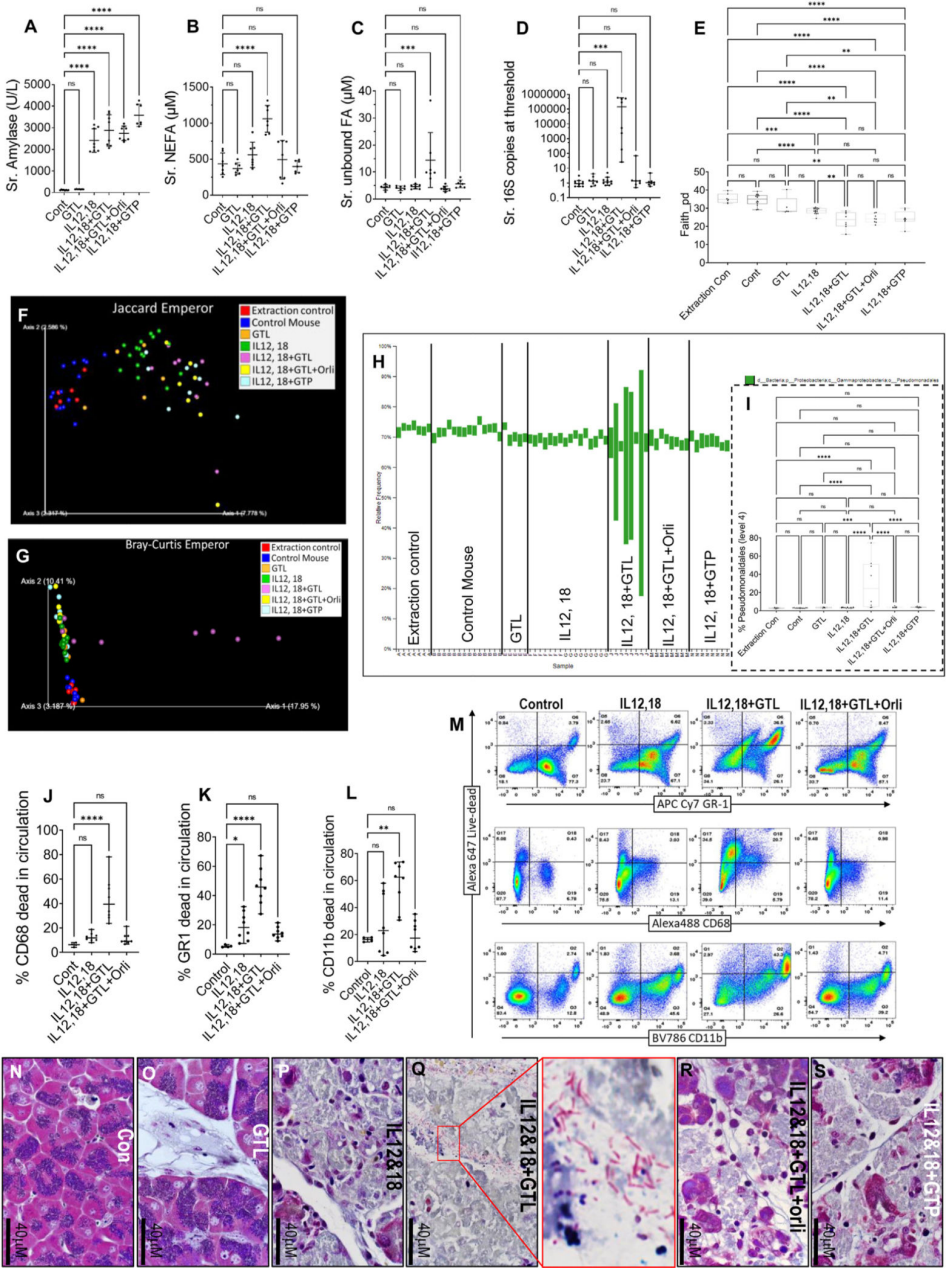
Effect of lipotoxic exacerbation of mouse IL12,18 pancreatitis on circulating lipids, microbiome, and immune cell injury: *Scatter plots* of different groups comparing serum amylase (*A*), NEFAs (*B*), unbound NEFAs (*C*), 16S bacterial DNA copy number (*D*), faith *α*-diversity (*E*), *β*-diversity by Jaccard-Emperor (*F*), Bray-Curtis Emperor (*G*) *plots*, and percentage of *Pseudomonadales* (*H*) in level-4 taxa *bar plots*, which are quantified and compared in *panel* (*I*). *Box plots* of flow cytometry data comparing percentage annexin V–positive CD68^+^ (*J*), GR-1^+^ (*K*), and CD11b^+^ (*L*) cells among different groups, and corresponding scattergrams (*M*), with each *row* corresponding to the lineage marker (*x-axis*) and *y-axis* for intensity of live-dead marker staining. (*N*–*S*) Gram-stained images (100×) of pancreas. Group is mentioned on *right*. (*Q*) Mixed flora bacteria (*zoomed inset*).

As reported previously,^9,25^ IL12,18-induced pancreatitis increased serum amylase in all groups (Figure 5A). This was like patients with AP irrespective of infection (Table 1). AP induced lipolysis of glyceryl trilinoleate (GTL [triglyceride of LA]) in the IL12,18+GTL group, which increased serum NEFAs (Figure 5B) and unbound NEFAs (Figure 5C) consistent with GTL lipolysis. This mirrored infected patients (Figure 1A and C). IL12,18+GTL group’s serum microbiome had higher 16S copies (Figure 5D), like infected AP (Figure 1D). These changes were absent in the IL12,18+glyceryl tri-palmitate group. Orlistat prevented the IL12,18+GTL–induced increase in serum NEFAs, unbound NEFAs, and 16S DNA copy number (Figure 5B–D). All pancreatitis groups had a reduction in *α*-diversity vs controls (Figure 5E). Similarly, *β*-diversity on the Jaccard-Emperor plot was different in all pancreatitis groups vs controls (permutational multivariate analysis of variance, <0.001) (Figure 5F). These changes in diversity are similar to patients with AP (Figure 1E and G) and suggest that pancreatitis, irrespective of infection, alters the circulating microbiome by unknown mechanisms. However, mice given IL12,18+GTL also had a further reduction in *α*-diversity compared with IL12 and IL18 alone (Figure 5E), suggesting enrichment of certain types of bacteria. Interestingly, the Bray-Curtis plot showed the IL12,18+GTL groups to be dissimilar from the rest (Figure 5G, permutational multivariate analysis of variance, <0.001), likely because this calculates on the basis of abundance, unlike the Jaccard distance, which is based on presence or absence. Level 4 taxa analysis showed IL12,18+GTL AP, unlike other groups uniquely increased *Pseudomonadales* (Figure 5H, *green bars* and 5I) similar to our patients with AP (Figure 1H).

To mechanistically understand the microbiome changes and higher bacterial loads (Figure 5E), we compared live dead marker staining in the circulating white cells with and without orlistat, which reduces NEFA and unbound FA levels (Figure 5B and C). The IL12,18+GTL group had a large increase in the death of CD68^+^, GR-1^+^, and CD11b^+^ cells, which were prevented by orlistat (Figure 5J–M and Supplementary Figure 11). GTL+IL12,18 induced death of approximately one-half of the circulating white cells (Supplementary Figure 12), which was again prevented by orlistat. Pancreatic Gram stain of mice given IL12,18+GTL showed polymicrobial flora (Figure 5Q, *zoomed inset*). Cytokines, including tumor necrosis factor–*α*, IL10, IL6, and MCP-1, that increased in infected human AP (Figure 1Q–U) were normalized by preventing an unbound FA increase in all of the rodent models (Supplementary Figure 13). Thus, infection and increased circulating bacteria during AP (Figures 1D, 4E, and 5D) may result from unbound NEFAs impairing bacterial clearance by means of amphipathic liponecrosis (Figures 1L-Q, 4F–K, 4R–V, and 5J–M).

## Discussion

**Figure 6.**
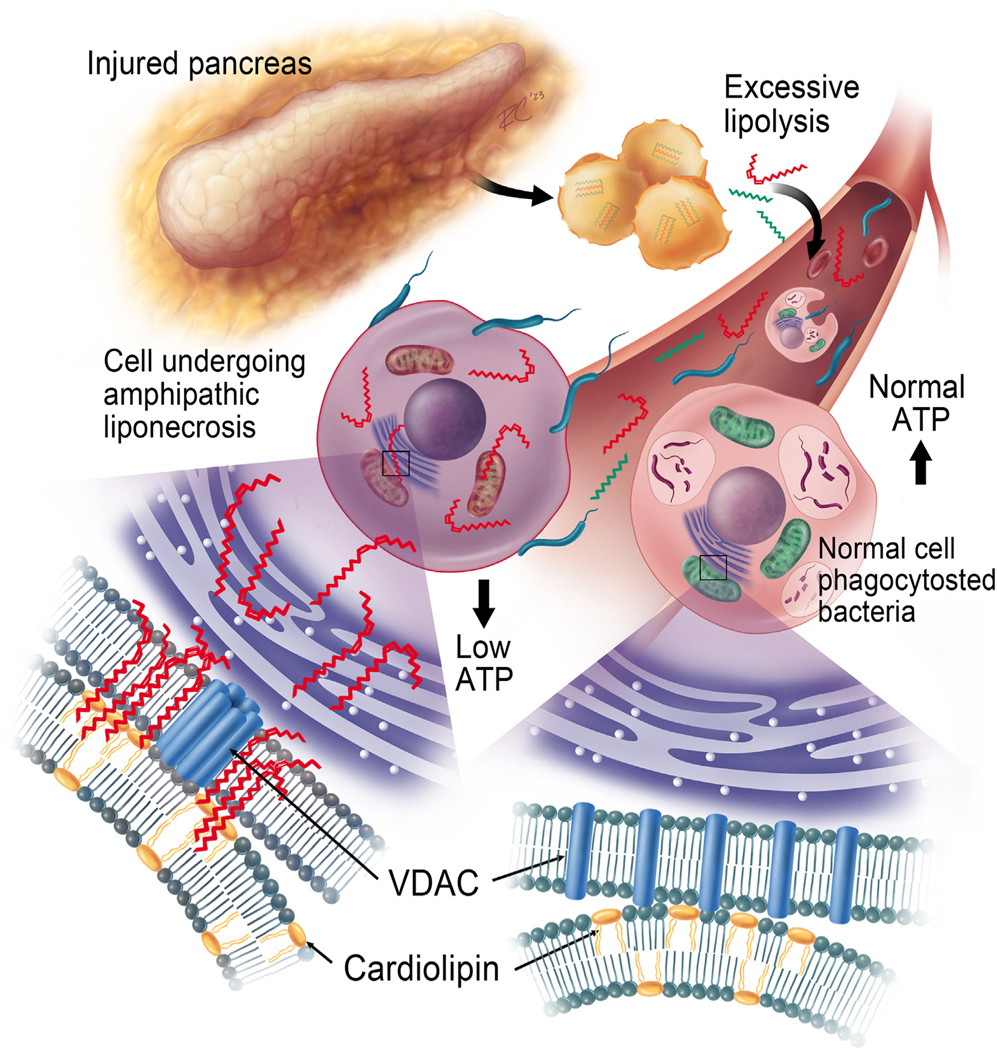
Model of unbound NEFAs mediating amphipathic liponecrosis–induced immune paralysis: During rapid lipolysis, such as in pancreatitis, the excessive release of NEFAs (shown as *zig-zag lines*) increases their unbound levels in the circulation and uptake into immune cells (*left cell*) via amphipathic interactions with membrane phospholipids. The consequent amphipathic liponecrosis involves interaction of these intracellular NEFAs with mitochondrial cardiolipin (*yellow*), enriching the unbound NEFAs in mitochondria and causing the normal VDAC monomers (*blue*) to oligomerize (note VDAC hexamer on *left side*). This, along with reduced ATP (from complex I, V inhibition, mitochondrial injury^9^), impedes cell functions like phagocytosis, reduces bacterial clearance and increases bacterial loads, resulting in infection.

We noted that the excessive release of amphipathic long-chain unsaturated OAs (C18:1) and LAs (C18:2) from lipolysis of visceral triglyceride pools can occur in mice or humans. Concurrent hypoalbuminemia^24^ in AP (Table 2) increases circulating unbound NEFAs to the low micromolar range (Figure 1C and J). In aqueous environments, these amphipathic unbound NEFAs have progressively favorable interactions with cell membrane phospholipids and mitochondrial cardiolipin, resulting in their cellular entry and eventually mitochondrial uptake. This induces VDAC oligomerization, mitochondrial depolarization, and low ATP levels; thus, impairing phagocytosis in macrophages (Figures 2 and 3) and causing inflammatory cell death. This increases bacterial loads and causes abdominal infections in all 3 in vivo models, irrespective of rodent species or pancreatitis (Figures 4 and 5). Sterile to septic transition, therefore, may occur via this amphipathic liponecrosis (Figure 6).

Although bacteremia, and therefore bacterial translocation, is common after dental and endoscopic procedures,^1,2,33^ such bacteremia is normally transient and does not result in infections or sepsis. This is consistent with translocated bacteria being cleared by competent immune cells, similar to our control and patients with noninfected AP and control mice given intraperitoneal GFP *E coli* (Figure 4C–K). Therefore, normally bacterial translocation^1,2,33^ is cleared, similar to our patients with noninfected AP, who, despite a change in the circulating microbiome (Figure 1G and H), did not develop infections. Our study, therefore, focused on the mechanisms that impair bacterial clearance and result in infections during pancreatitis. The infections we noted (Table 2) include bacteremia, lung infections, abdominal and pancreatic infections, and urinary infections. This pattern of infections agrees with the high prevalence of extrapancreatic infections (30%–80%) reported in AP.^4,5,34,35^ Interestingly, *Pseudomonadales*, which include *Acinetobacter* and *Pseudomonas,* comprised 30%–40% of the microbiome of our patients with AP (Figure 1H) and IL12,18+GTL mouse microbiome (Figure 5H and I). These findings are similar to those of Ni et al, who found *Acinetobacter* comprised 26% and *Pseudomonas* comprised 8% of isolates in human AP, and also agreed with those of Lu et al,^4^ wherein *Pseudomonas* and *Acinetobacter* were the most common organisms isolated in AP.

We found that unbound NEFA–induced amphipathic liponecrosis impairs bacterial clearance. Amphipathic liponecrosis by unbound NEFA differs from albumin-bound NEFA signaling as: 1) being transport/receptor protein–independent (Figure 2B, G, and H)^36^; 2) being rapid onset (in seconds; Figure 2A–J) vs *β*-oxidation (in minutes^37^); 3) localizing to membranes (Figure 2F–H), while lipid droplets are cytoplasmic; 4) injuring mitochondria^9,38^ and reducing ATP (Figure 3D, E, and M), unlike albumin-bound NEFAs, which increase ATP (Figure 3M, *third bar*); and 5) early steps being reversible by albumin (Figure 2M). Therefore, the acute lipotoxicity mediated by unbound NEFAs that results in amphipathic liponecrosis is mechanistically and pathophysiologically distinct from uptake of albumin-bound FAs.

Although we focused on VDAC oligomerization, other mitochondrial changes during LA lipotoxicity include mitochondrial complexes I and V inhibition, mitochondrial swelling, inclusions, loss of cristae, cytochrome C leakage,^9,38^ with reduced ATP and necrosis.^9^ Similarly, uncoupling proteins may mediate the loss of ψm by unbound NEFAs.^39^ Betaneli et al^29^ previously reported that cardiolipin, with its bulky 4 acyl chains, prevents VDAC oligomerization. We thus hypothesized that VDAC oligomerization results from unbound LA monomers in internal membranes that interact with mitochondrial LA-cardiolipin and reduce the distance maintained by cardiolipin between the VDAC monomers, thus leading to VDAC oligomerization. The consequent immune cell “amphipathic liponecrosis” (Figure 6) may thus explain how sterile diseases like pancreatitis can become infected.

In human AP, the generic increase in 16S DNA and altered *α*- and *β*-diversity (Figure 1D and G) are consistent with transient bacteremia after dental extractions^1^ and endoscopic procedures.^2,33^ Patients with noninfected AP with lower unbound NEFAs (Figure 1C and J) and normal inflammatory cells (Figure 1L–P) clear these bacteria similar to resolution of transient bacteremia^1^ or uncomplicated diverticulitis without antibiotics.^40^ Higher 16S in infected AP correlate with higher unbound NEFAs (Figure 1C and D) because >2–10 *μ*M OA and LA may injure immune (CD3^+^ and CD14^+^)^10^ and splenic white cells (Figure 1K–P). This immune cell injury may also synergize with immunesuppression from tumor necrosis factor–*α* and IL10,^41^ the levels of which increase with lipotoxicity (Supplementary Figure 13).

Serum unbound OA and LA during infection (2–40 *μ*M) are below^25^ their respective critical micellar concentrations of approximately 40 *μ*M and 160 *μ*M at 37°C (pH 7.4). The lower uptake, ψm, higher ATP for OA vs LA (Figures 2I–J and 3D) at similar concentrations is consistent with their critical micellar concentrations. However, both LA and OA achieve serum concentrations to cause multisystem injury^24^ (Figures 4 and 5). PA’s critical micellar concentration is <8 *μ*M, which explains PA’s inability to increase circulating NEFA^24^ (Figures 4A and 5B), its low cellular uptake, ψm (Figure 2I–J), and minimal VDAC oligomerization (Figure 3C), despite using higher PA concentrations and a dimethyl sulfoxide solvent. In addition, PA in triglycerides interferes with the triglyceride’s lipolysis.^25^ These mechanisms explain the patterns of immune cell injury induced by different NEFAs (Figures 4A–K and 5A–I).

Visceral fat comprises 2%–10% of body weight,^42^ with >80% of adipose mass being triglyceride.^43,44^ We use triglycerides of LA (GTL) or PA (glyceryl tri-palmitate)^25^ because human visceral fat is mostly unsaturated.^8,45^ Although both obesity^9,11,13^ and triglyceride administration^10^ worsen AP outcomes similarly,^25^ the use of triglycerides bypasses the chronic changes (eg, insulin resistance, baseline adipose inflammation, weight gain, and adaptive changes) associated with increasing visceral fat due to obesity.

Although the number of cell lineage markers we study on flow cytometry is limited, our modeling of sterile to infected transition is clinically relevant because most sepsis starts sterile^3^ and septic patients injure splenic lymphocytes^16,17^ or have lymphopenia,^16,17^ similar to our findings. Moreover, increased lipase activity has previously been noted in critical illnesses^46^ and sepsis.^47^ Therefore, the lipolytic release of excessive NEFAs could potentially cause immune paralysis in other diseases with painless or subclinical pancreatic injury.^48^

In summary, we report that excessive lipolytic release of LA and OA from visceral triglyceride lipolysis can cause immune cell mitochondrial injury by uptake via an amphipathic interaction between unbound NEFAs and membrane phospholipids. This can impair bacterial clearance and thus convert a sterile disease like pancreatitis to an infected disease. Whether this phenomenon causes sepsis in other sterile diseases remains to be seen.

## Supplementary Material

1

2

Video 1

Video 2

Video 3

Video 4

Video 5

Video 6

Video 7

Video 8

Video 9

## Figures and Tables

**Table 1.** Demographic, Clinical, and Biochemical Characteristics of Patients Included in the Study: Comparison of Patients With Acute Pancreatitis Sampled for Microbiome Analysis (Sampled Acute Pancreatitis) With Normal Controls and Other Patients With Acute Pancreatitis Admitted During the Same Period

Parameter	Normal controls (n = 53)	All AP (n = 199)	Sampled AP (n = 62)	*P* value
Age, *y*, mean ± SD	54.7 ± 13.4	55.8 ± 16.6	58.6 ± 16.0	.20
Sex, *female/male*, n	14/39	80/119	19/43	NS
Body mass index, *kg/m^2^*, mean ± SD	28.8 ± 4.6	29.5 ± 5.6	29.5 ± 6.4	.78
Etiology of AP, n	—			
Biliary	—	49	23	.45
Alcohol	—	52	15	.85
Hypertriglyceridemia	—	8	4	.71
Endoscopic retrograde cholangiopancreatography	—	10	4	.76
Other	—	28	11	.37
Idiopathic	—	52	16	.31
Admission parameter, serum lipase, *U/L*, mean ± SD	24 ± 17	1249 ± 1156^a^	1162 ± 1063^a^	<.001
Admission lipid parameters, mean ± SD				
Serum NEFA, *μM*	297 ± 155	832 ± 540^a^	830 ± 578^a^	<.001
Unsaturated NEFA, %	55.6 ± 5.7	60.6 ± 5.4^a^	59.7 ± 5.7^a^	<.001
Unsaturated NEFA, *μM*	171 ± 99	514 ± 358^a^	519 ± 405^a^	<.001
Unbound NEFA, *μM*	1.0 ± 0.6	3.6 ± 5.1^a^	4.8 ± 8.2^a^	<.001

a Significantly different from controls.

**Table 2.** Demographic, Clinical, and Biochemical Characteristics of Patients Included in the Study: Comparison of Patients With Infected Acute Pancreatitis With Those Without Infection

Parameter	Noninfected AP (n = 41)	Infected AP (n = 21)	*P* value
Age, *y*, mean ± SD	55.9 ± 16.8	65.1 ± 14.1	.06
Sex, *female/male*, n	13/28	6/15	.8
Body mass index, *kg/m^2^*, mean ± SD	30.0 ± 6.9	29.3 ± 6.1	.68
Etiology of AP, n			
Biliary	14	8	.41
Alcohol	12	3	.53
Hypertriglyceridemia	2	2	.56
Endoscopic retrograde cholangiopancreatography	2	2	.56
Other	0	1	.34
Idiopathic	11	5	1.0
Laboratory parameters, mean ± SD			
Serum lipase, *U/L*	1144 ± 1011	1208 ± 1112	.82
Serum albumin, *g/dL*	4.4 ± 0.5	3.7 ± 0.9^a^	<.02
Outcomes			
Length of stay, *d*, mean ± SD	3.4 ± 3.0	18.3 ± 20.5^a^	<.003
Patients with sepsis, %	0	13^a^	<.001
Microorganism isolated, %	0	13^a^	<.001
Antibiotics for AP, n (%)	0 (0)	21/21^a^	<.001
Sites of infection, n			
Blood	—	6	—
Lungs	—	6	—
Abdomen	—	5	—
Urinary tract	—	2	—
Lipid parameters, *μM*, mean ± SD			
Serum NEFA	670 ± 288	1196 ± 784^a^	.013
Unbound NEFA	2.6 ± 1.2	6.9 ± 4.1^a^	.02

a Significantly different from noninfected AP group.

## WHAT YOU NEED TO KNOW

### BACKGROUND AND CONTEXT

Although transient bacteremia commonly occurs with dental and endoscopic procedures, acute pancreatitis, like other sterile illnesses, may get infected by unknown mechanisms.

### NEW FINDINGS

Albumin-unbound fatty acids injure immune cells by interacting with cell membrane phospholipids, enriching in mitochondrial membranes, and impairing bacterial clearance, thus, causing infection and sterile to septic transition.

### LIMITATIONS

Human samples only included blood samples at admission and not later in the disease. A limited panel of immune cell lineage markers were studied for immune cell injury in vivo.

### CLINICAL RESEARCH RELEVANCE

Albumin unbound nonesterified fatty acids (NEFAs) may cause similar sterile to septic transition in other hypoalbuminemic states that have elevated fatty acids, including liver disease, sepsis, burns, cardiovascular events, and critical illnesses.

### BASIC RESEARCH RELEVANCE

Long-chain, unsaturated, unbound NEFAs are aqueous stable at low micromolar concentrations and enter cell membranes by interacting with membrane phospholipids independent of proteins, which is reversible by albumin in early stages. Within cells, these unsaturated, unbound NEFAs cause mitochondrial injury by enriching in mitochondrial membranes, which have cardiolipin that interacts favorably with the unbound NEFAs; this impairs immune cell functions like phagocytosis.

## Abbreviations used in this paper:

AP: acute pancreatitis
AU: arbitrary units
CER: caerulein
FA: fatty acid
GTL: glyceryl trilinoleate
GTO: glyceryl trioleate (triolein)
GTOO: glyceryl trioleate D orlistat
IL: interleukin
ITC: isothermal titration calorimetry
LA: linoleic acid
MROS: mitochondrial reactive oxygen species
NEFA: nonesterified fatty acid
OA: oleic acid
PA: palmitic acid
PBS: phosphate-buffered saline
VDAC: voltage-dependent anion channel
