# Supplementary material for: Amphipathic Liponecrosis Impairs Bacterial Clearance and Causes Infection During Sterile Inflammation
Source: Gastroenterology. Author manuscript; Available in PMC 2023 Dec 7. (PMC10703425; doi:10.1053/j.gastro.2023.05.034)
Supplement: 1 [file NIHMS1905536-supplement-1.pdf]

SUPPLEMENTARY METHODS:

Autopsy studies: These were approved by the Mayo Clinic Institutional Review Board; informed consent was waived. The Mayo Clinic tissue registry was searched from 1995 to 2015 for patients who had an antemortem diagnosis of pancreatitis and postmortem diagnosis of necrotizing pancreatitis at the time of autopsy. 16 such decedents were retrieved: 9 had undergone debridement at the time of hospitalization, the fluid/tissue of which was cultured, and these had documented infected necrosis (infected necrosis group), while the other 7 had necrotizing pancreatitis without documented infection (non-infected group). Sections were available for 6 spleens from the 9 in the infected necrosis group and for 4 spleens in the non-infected necrosis group. These are detailed in table 1A. To compare the effect of pancreatitis vs. unrelated causes of death, 9 control patients who had an autopsy after dying from an unrelated cause were randomly chosen and retrieved (Table 5B). These control autopsies were done at our institution during the same period when the pancreatitis autopsies were done. These were chosen in the order they presented, based on availability of tissues for study, till the required number to match the pancreatitis cases was achieved. Exclusion criteria included those receiving chemotherapy, a diagnosis of pancreatitis, and ensuring that the control patient did not present with a sterile disease that later became septic. Spleen (n=8) sections were available for some cases. There was no significant difference in the period between death and accession for autopsy between control and AP groups (5.9±3.0 vs. 10.3±6.2 hours, $p=0.11$).

Fluorescent immunohistochemistry staining: Formalin fixed paraffin embedded (FFPE) sections of spleen were rehydrated through xylene and graded alcohols followed by heat-induced epitope retrieval in IHC-Tek Epitope Retrieval Solution (IHC World, LLC, Woodstock MD) in a Black & Decker Handy Steamer model HS1000 (Black & Decker, Townson, MD) at approximately 95^0C for 40 minutes followed by a 20 minutes of cooling on the benchtop at room temperature. Endogenous peroxidase was quenched in 3% hydrogen peroxide at room temperature for 15 minutes followed by blocking in 5% BSA in 0.1% TBS-T for one hour. Cleaved, active caspase-3 (Asp175) was probed with a rabbit polyclonal antibody (1:200 dilution; #9661, Cell Signaling Technology, Inc, Danvers, MA) overnight at 4 ˚C followed by highly cross-adsorbed anti-rabbit secondary conjugated to CF647 (1:500 dilution; Biotium, Inc, South San Francisco, CA) for 1-2 hours at room temperature. Nuclei were counterstained with 1 µg/mL DAPI (ThermoFisher Scientific, Grand Island, NY) and mounted with Fluoromount G (Sigma Aldrich, St. Louis, MO) and sealed with nail polish (Quick-Dry Top Coat, Revlon, New York, NY).

Images were collected on a Zeiss AxioObserver.Z1 inverted microscope and a Zeiss AxioCam 506 mono 6-megapixel CCD camera run by Zeiss Zen 2 Pro software (all from Carl Zeiss Microscopy, LLC, Thornwood, NY). Illumination was provided by a Lambda XL broad spectrum light source (Sutter Instrument, Novato, CA) with appropriate filters for DAPI, FITC, TRITC and Cy5 (Chroma and Semrock). Images were collected with a Zeiss C-Aprochromat 63x/1.2 NA water immersion objective or a Plan-Apochromat 20x/0.8 NA air objective.

Automated image cytometry to quantify percent caspase-3 positive cells was performed with ImageJ/FIJI. Briefly, nuclei in the DAPI channel were segmented by auto-thresholding followed by a binary watershed to separate adjacent nuclei. Individual nuclei were identified as primary objects by the Analyze Particles module and positions were stored in the ROI Manager module. Caspase-3 signal was detected by dilating the stored ROI position for the nuclei and measuring the mean signal intensity for each nucleus in the Cy5 channel. Results were exported to an Excel Spreadsheet and statistics were performed in GraphPad Prism (Version 7; GraphPad Software, La Jolla, CA).

MICROBIOME STUDIES:

Handling of serum, plasma samples: The serum samples were stored in sterile containers at 4^0C and transported to the laboratory on ice within 24 hours where they were aliquoted immediately and frozen at -80^0C till further use. All plasticware used for handling samples was sterile, DNA, RNA, DNAse free, with barrier tips. Samples were always opened using mask, gloves, and behind a transparent shield using standard microbiology, and molecular biology precautions. All subsequent usage was with ≤2 freeze thaw cycles.

Cell-free DNA isolation: Cell-free DNA (cfDNA) was isolated from plasma or serum as per user guide of MagMAX Cell-free DNA isolation kit (Thermo Fisher Scientific, Waltham, MA, USA). In brief, human or mouse

serum or PBS (~100µl) was mixed with MagMAX Cell-free DNA Magnetic beads (10µl), and binding solution (400µl). The mixture was vortexed thoroughly (10min, 25ºC), placed the tubes on the DynaMag Magnet (5 min, 25ºC) to separate beads from the supernatant, and carefully discarded the supernatant. The beads were washed with wash solution thrice, and with ethanol (80%) twice. The beads were dried up, and cfDNA was eluted out from the bead with ultrapure water (10µl). The concentration of the DNA in the eluent was measured by NanoDrop Microvolume Spectrophotometers (Thermo Fisher Scientific, MA, USA), and processed for qPCR assay. Sterile PBS (2 samples/ batch of 10-14 samples) served as extraction controls in place of serum to assess background noise for the cell free DNA extraction. Each batch had samples from all patient groups. The extraction controls determined the threshold cycles to compare and quantify the 16S bacterial DNA amounts using primers corresponding to V3-V4 hypervariable regions (please see below) for each batch. The cell free DNA was isolated from human samples in 7 batches.

Amplification: PCR reaction was performed with the universal 16S bacterial primer pair 5'-CCTACGGGNGGCWGCAG-3', and 5'-GACTACHVGGGTATCTAATCC-3' as recommended[1]. In a qPCR plate, the reaction volume (20µl) was prepared with SYBR Green Master Mix (10µl), F-primer (1µl), R-primer (1µl), cfDNA (1µl), and ultrapure water (7µl). The following PCR conditions were used, and run on the LightCycler 480 II instrument (Roche, Indianapolis, IN, USA): Initial denaturation at 95ºC for 5 min, followed by 40 cycles consisting of denaturation (95ºC for 40 s), annealing (55ºC for 2 min) and extension (72ºC for 1 min) and a final extension step at 72ºC for 7 min. The 16S copy number was calculated from the qPCR cycle number after subtracting extraction control's cycle number.

Microbiome library preparation methodology: This was done in the genomics core facility at Arizona State University. Bacterial community analysis was performed via next generation sequencing in MiSeq Illumina platform. Amplicon sequencing of the V4 region of the 16S rRNA gene was performed with the barcoded primer set 515f/806r designed by Caporaso et al[2] and following the protocol by the Earth Microbiome Project (EMP) (http://www.earthmicrobiome.org/emp-standard-protocols/) for the library preparation. PCR amplifications for each sample were done in triplicate, then pooled and quantified using Quant-iT™ PicoGreen® dsDNA Assay Kit (Invitrogen, Waltham, MA). A no template control sample was included during the library preparation as a control for extraneous nucleic acid contamination. 240 ng of DNA per sample were pooled and then cleaned using QIA quick PCR purification kit (QIAGEN, Germantown, MD). The pool was quantified by Illumina library Quantification Kit ABI Prism® (Kapa Biosystems, Wilmington, MA). Then, the DNA pool was diluted to a final concentration of 4 nM then denatured and diluted to a final concentration of 4 pM with a 15% of PhiX. Finally, the DNA library was loaded in the MiSeq Illumina and run using the version 2 module, 2x250 paired-end, following the directions of the manufacturer.

Microbiome bioinformatics: Microbiome bioinformatics were performed with QIIME2-2021.11 as described by Boylen et al[3]. Raw sequence data were demultiplexed using the q2-demux plugin adapters and primers were removed by cutapat plugin (DOI:10.14806/ej.17.1.200, https://cutadapt.readthedocs.io/en/stable/) followed by denoising and duplicates removal with DADA2 (via q2-dada2)[4] which generating amplicon sequence variants (ASVs). Vsearch consensus method was used for feature classification (https://www.ncbi.nlm.nih.gov/pmc/articles/PMC5075697/) against Sliva database(https://www.arb-silva.de/; version 138-99), ASVs were aligned with mafft[2] (via q2-alignment) and used to construct a phylogeny with fasttree2 (via q2-phylogeny)[5]. Alpha-diversity metrics (observed features and Faith's Phylogenetic Diversity[6]), beta diversity metrics (weighted UniFrac[7]), unweighted UniFrac[8], Jaccard distance, and Bray-Curtis dissimilarity), and Principle Coordinate Analysis (PCoA) were estimated using q2-diversity after samples were rarefied (subsampled without replacement) to 10000 sequences/sample for 16S rRNA gene, respectively. The Bray-Curtis dissimilarity is based on occurrence data (abundance), while the Jaccard distance is based on presence/absence data (does not include abundance information). For determining mitochondrial DNA, Fastq sequences were mapped to human genome reference HG38 by Burrow-Wheeler Aligner (BWA), we counted reads mapped to human genome reference as well as that mapped human mitochondria for each sample. To evaluate mitochondria damage, we used % mitochondria read over that for total reads.

Quality Control of microbiome studies:

1) <u>Steps to minimize bacterial contamination</u>
   A) Samples were collected, transported (4^0C) and stored (-80^0C) in sterile containers.
   B) All plasticware used for handling samples was sterile, DNA, RNA, DNAse free, with barrier tips.
   C) Samples were always opened using mask, gloves, and behind a shield using standard microbiology, and molecular biology precautions.
2) <u>Using extraction controls as negative controls for each serum batch, and their post-hoc analysis</u>; These controls were present at all steps from cell free DNA extraction till microbiome analysis:
   A) Sterile PBS (2 samples/ batch of 10-14 samples [now mentioned in the suppletory section]) served as extraction controls in place of serum to assess background noise for the cell free DNA extraction, and amplification. Each batch had samples from normal controls, non-infected and infected patients.
   B) Extraction control noise is a reference for all microbiome data. For e.g. in Fig 1D, the "threshold" for copy number is determined by extraction controls. This was typically at 26 cycles.
   C) On quantification, the extraction controls in Fig 1H, and Fig 5H, I *(Pseudomonadales*) are no different from the normal patient controls. This reduces probability of contamination in control sera. Therefore the significantly higher signal of *Pseudomonadales* in the AP patient samples in these figures are not contamination, since these were processed in parallel.
   D) similar to Schierwagen et al[9], we have provided beta-diversity plots including the extraction controls in Figure 5F, and 5G. We note again that the extraction controls in these are the same as in control mice sera (note the intermingling of blue and red dots). However, the blue and red clusters are clearly separated from the rest.
   E) We note that 13/14 human extraction controls (green dots in white dashed outline) are separated from the pancreatitis patients (red and yellow dots shown to the right, with PERMANOVA <0.0001 for extraction controls vs. other groups.

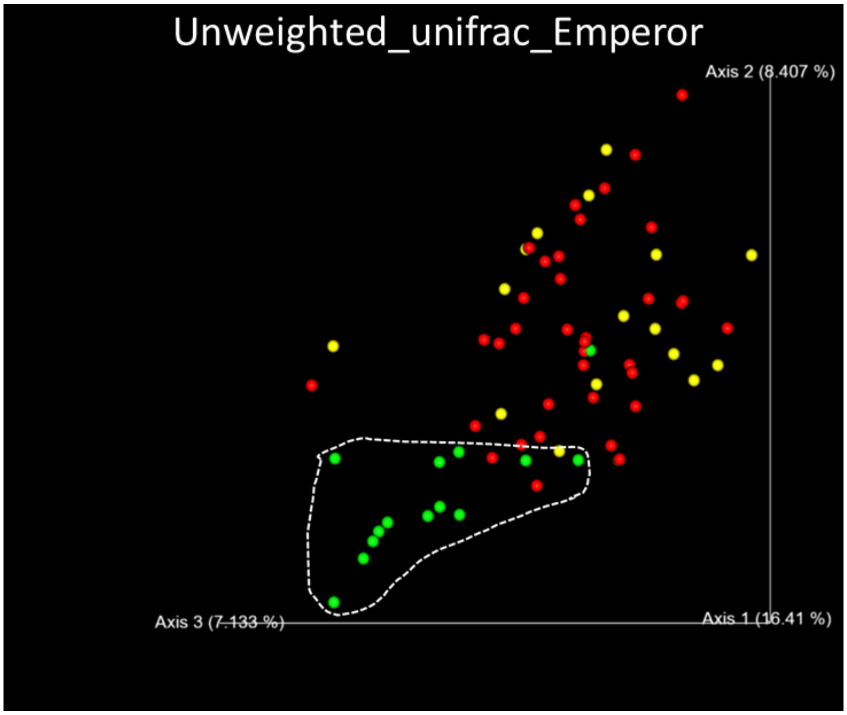

3) <u>Using external reference as positive controls</u>:
   A) blood cultures were done in 6 of the infected 21 patients. The positive blood cultures served as external positive controls. The 3 positive blood cultures identified the same organisms that were enriched in microbiome analysis. This is mentioned in the second section of results as: Three among 6 blood cultures collected at the same time were positive for bacterial growth in the infected group. These 3 had a high proportion of the cultured organism on microbiome analysis (% in parenthesis) including *Escherichia coli* (21.7%), *Clostridium perfringens* (81%) and *Cronobacter* (14%), *Staphylococcus aureus* (12%).

4) <u>Comparing identified organisms to published literature:</u> The main types of organisms in our infected patients (ie. *Pseudomonadales* including *Acinetobacter and Pseudomonas;* shown in figure 1H and the Linear discriminant analysis in Supplementary Fig 2) are similar to what others have identified by cultures of infected AP patients. For e.g., we identify Pseudomonadales to average at ≈30% of our microbiome (fig 1H; B in image below). similarly, Li et al show *Acinetobacter* being the highest prevalence at 26% and *Pseudomonas* at 8%. Additionally, Lu et al[10] identify *Pseudomonas* and *Acinetobacter* as the most common organisms isolated. Please see comparison to their table below: Therefore, these organisms are present early in AP, but are cleared by the competent immune cells in the group that does not develop infections, while the group with immune cell injury and impaired clearance develops infections.

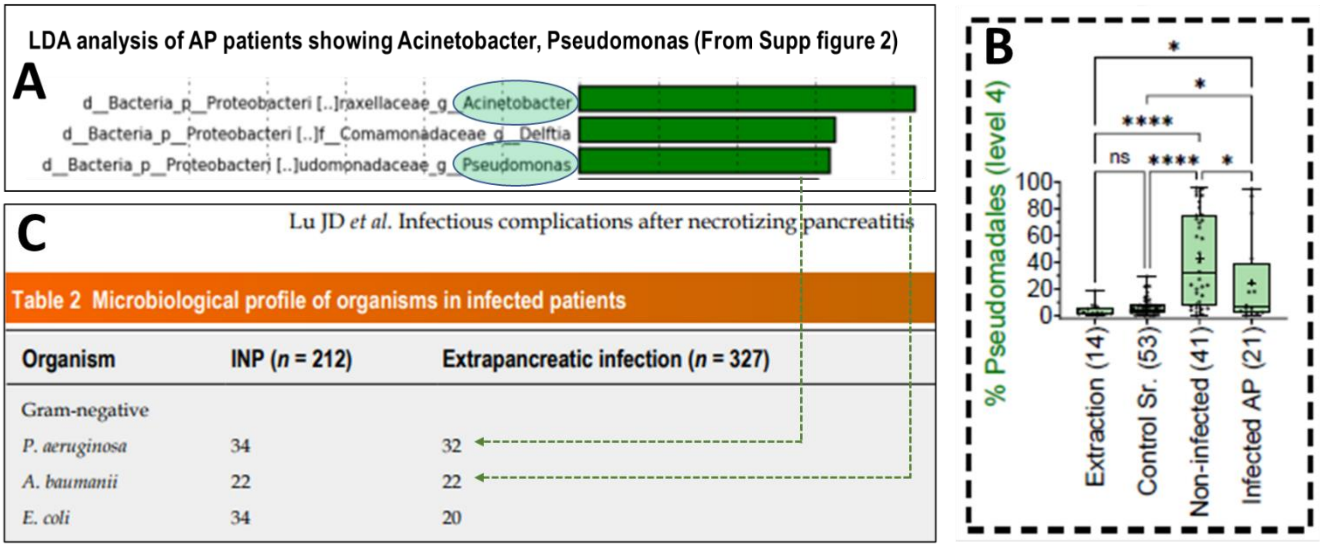

Organism	INP (*n* = 212)	Extrapancreatic infection (*n* = 327)
Gram-negative		
P. aeruginosa	34	32
A. baumanii	22	22
E. coli	34	20

Comparison of the organisms we identified in infected AP patients shown as enrichment of Acinetobacter and Pseudomonas by LDA analysis (A; from supplemental figure 2), and as proportion of Pseudomonadales (B; from Figure 1H). Note enrichment of Pseudomonadales in AP patients compared to extraction controls and normal control serum. C: Table from Lu et al showing high prevalence of Pseudomonas and Acinetobacter in infected AP.

ANIMAL STUDIES:

All experiments were approved by the Institutional Animal Care and Use Committee of the Mayo Clinic; care and handling of animals were in accord with the Guide for the Care and Use of Laboratory animals by the Institute for Laboratory Animal Research.10-12 weeks old male CD1 mice (Charles River Laboratories, Wilmington, MA) or C57BL/6J mice (The Jackson Laboratory, Bar Harbor, ME) were acclimatized for at least 2 days before experimentation. Mice were housed at temperatures ranging from 21-25 °C with a 12-hour light/dark cycle, fed standard laboratory chow and allowed to drink *ad libitum*. 200-300 gm male Wistar rats were purchased from Charles River laboratories (Wilmington, MA) were acclimatized and fed in a similar fashion.

Acute Pancreatitis models: To induce pancreatitis, the murine recombinant IL-12 (Peprotech, Cranbury, NJ, 150 ng/30g) and IL-18 (R&D Systems, Minneapolis, MN, 750 ng/30g) were injected into peritoneal cavity, two consecutive doses at 24h intervals as described previously[11]. A single dose of 150µl (0.5% body weight) glyceryl trilinoleate (GTL, TCI Inc, Philadelphia, PA), GTP (Sigma Aldrich; surgically placed 150 mg powder) or GTL+orlistat (Cayman Chemical, Ann Arbor, MI, 50 mg/kg dissolved in GTL) was given intraperitoneally 2 hours after the second dose of IL12&18 as described previously[11]. Animals were followed for 3 days after the second injection of IL12&18 or till they were moribund, which ever came first, at which time they were euthanized in a carbon dioxide chamber before harvesting blood and tissues. 90% mice in the IL12&18+ GTL group became moribund between 48-72 hours after the second IL12&18 injection as shown previously[11]. Cerulein AP was induced in rats as previously[12] by intraperitoneal caerulein injection (20 mcg/kg 2 times a day) for 3 days. For rats co-administered the triglyceride of oleic acid (glyceryl trioleate; GTO or triolein; Sigma-Aldrich), GTO was given at 3% of body weight based on a previously optimized protocol[12] that simulates

human obesity. GTO alone, as shown previously[12] (see supplementary figure 5 of[12]) does not cause any deleterious effects or pancreatitis. In some rats with caerulein pancreatitis, the lipase inhibitor orlistat (Cayman Chemical, Ann Arbor, MI) was co-administered (at 50 mg/kg) dissolved in GTO (i.e. CER+GTOO group), with a goal to prevent lipolysis of GTO to oleic acid. Rats were followed for 3 days and sacrificed 3-4 hours after the last injection or when the CER+GTO group was noted to be moribund, which was typically on the third day (Fig 4Q).

Carotid pulse distension (MouseOx Oximeter, STARR Life Sciences, Pittsburgh, PA) was used as a measure for noninvasive blood pressure monitoring. Shock was defined as reduction tp <40% of baseline. Mice and rats were observed four times daily. Terminal blood and tissue harvest was done and used in the analyses. Tissues were collected for histology and TUNEL staining (formalin). Blood was collected for flow cytometry and processed as below. Serum for lipidomics, Luminex and biochemical assays (serum) was processed as described below and detailed previously[13, 14].

Intraperitoneal GFP-E coli and NEFAs injection and mimicking sepsis like model *in vivo*: CD-1 mice were used for this experiment. These were divided into different groups, i.e. control, GFP-*E. coli* (ATCC® 25922GFP™) alone, and GFP-*E. coli* given along with Oleic acid (OA, 0.3% body weight), and Palmitic acid (PA, 0.3% body weight). Additionally, Linoleic acid (LA, 0.2% body weight) +fatty acid free bovine serum albumin (BSA) or GFP-*E. coli* with LA ± Alb were injected into CD1 mice in different groups. For the LA+ albumin group LA was pre-conjugated with BSA and Calcium (60µl LA with 1.2 ml of 25% BSA and 20mM $CaCl_2$) at 37ºC for 2-4h. There were 8-12 mice per group. 10^6 GFP-E coli/mouse in 0.1ml saline were injected IP in the respective group of mice. After 30 min of GFP-E coli injection, pulse distension, and rectal temperatures were measured at baseline and daily thereafter. 14 hours later, pHrodo E. coli (10^6/mouse) were given intraperitoneally, and mice were sacrificed using carbon dioxide and the cells in the peritoneal fluid were stained with live dead marker. To quantitate bacterial load, ascitic fluid/peritoneal lavage was collected. Postmortem blood was collected for total WBC count, processed for flow cytometry from heparinized samples (as below) and plasma was collected for measuring blood urea nitrogen, albumin, Ca^{2+}, uFA. Kidney, lung, and spleen samples were formalin fixed; paraffin embedded. These were stained for TUNEL, using the method described below under immunofluorescence staining.

Peritoneal bacterial loads: To quantify bacterial loads the peritoneal cavity was lavaged with 1-2 ml saline at the time of sacrifice and the sediment was pelleted at 10,000g for 30 minutes. The pellet was resuspended in 1 ml saline, sonicated with a probe sonicator (15 Seconds), and centrifuged again at 10,000 g/10 min. Fluorescence was measured in the supernatant with excitation at 488nm and emission at 510 nm Flex Station 3 microplate reader (Molecular Devices, Sunnyvale, CA), and normalized to peritoneal washings from rodents to which no bacteria were administered. This was compared to a standard curve prepared with known amounts of E. coli GFP bacteria to quantify the bacterial load in the peritoneal cavity. The bacterial counts were confirmed by using a Neubauer chamber.

Gram's staining: This was done using the commercially available Kit from Sigma-Aldrich (St. Louis, MO) on deparaffinized formalin fixed paraffin embedded sections of the pancreas and surrounding fat of rodents used in the study. Imaging was done using an Olympus microscope with a 100x/1.3NA oil objective with a DP74 Digital Color Camera, and cellSens entry imaging software. Slides were scanned for bacteria using a 10x-40x objective. 2-4 representative images of bacteria or their absence were taken at 100x for each pancreas. Bacteria when present were usually in large numbers and multiple fields. Number of pancreas from each group that were positive for bacteria, along with type of bacteria were recorded.

Luminex studies for serum cytokines: These were done on human[15] and rodent serum samples[12, 14] as described previously.

Serum Biochemical and blood hematological assays and lipidomics assays:
Pancreatic lipase was assayed using the Kit (L7503) from Pointe Scientific (Canton, MI) as per protocol for the clinically established method. This uses a 1,2-diglyceride substrate, with colipase as the pancreatic lipase cofactor, and deoxycholate (1.0 mM) which acts both as an activator for pancreatic lipases and inhibitor of lipoprotein lipase. Serum amylase and blood urea nitrogen (BUN) were also assayed per clinically established methods using kits from Pointe Scientific using A7564 and B7552 respectively. Total peripheral WBC counts was done manually using a hemocytometer as per established protocols after lysing EDTA blood or

heparinized blood in 1% acetic acid. For serum fatty acids, the samples were aliquoted and stored at -80'C. Batch analyses for total serum NEFA were done using the Wako HR series NEFA-HR(2) enzymatic colorimetric method (Fujifilm, Valhalla, NY) as previously[11, 15]. Unbound fatty acid were measured using the Kit from FFA sciences (San Diego, CA) as described previously[11, 15].

TUNEL staining in Spleen sections: Spleen tissue from FFPE sections (5 μm) was used for terminal deoxynucleotidyl transferase dUTP nick end labeling (TUNEL). In brief, after deparaffinization, the TUNEL Andy Fluor™ 647 Apoptosis Detection Kit (GeneCopoeia, Rockville, MD) was used to detect DNA cleavage in apoptotic cells in tissue sections as per the mouseocmanufacturer's instructions. The staining was completed with fluorescent nuclear counterstain with Hoechst 33342 solution (1:1000, Thermo Scientific). Digital images of section were captured with a digital microscope Axio Imager. M2 or Axio Observer.Z1 (Zeiss, Oberkochen, Germany) and quantified as percentage of TUNEL positive nuclei in the spleen tissue.

Flow cytometry: Human samples collected in citrated and EDTA blood vials (after performing routine laboratory test) were used. RBCs were lysed in FACS lysing solution (BD Biosciences, CA, USA) for 15 min at room temperature. WBCs were washed with FACS staining solution (BD Bioscience, CA, USA) three times at 1,600 rpm for 5 min at 4^0C. Approximately, 1 x 10^6 cells were suspended in FACS staining solution (100 μl/tube) having 1mM $CaCl_2$ since annexin-V and phosphatidylserine binding is calcium dependent. The cells were incubated with a mixture of Annexin V-Alexa Fluor 488 (Thermo Fisher Scientific, MA, USA) and different cell lineage markers (Brilliant Violet 711 anti-human-CD3 and APC/Cy7 anti-human-CD14) from Bio-legend, CA, USA for 45 min at 4^0C in dark. Then excess antibodies were washed off two times with FACS staining solution and finally cells were suspended in same buffer (500 µl) for acquisition. Annexin V positivity of total, CD3 sub-population, and CD14 sub-population were analyzed. Similarly, Brilliant Violet 711™ Mouse IgG2a kappa and APC/Cy7 Mouse IgG1 kappa also from Biolegend, CA, USA were used for respective isotype control of CD3 and CD14.

Flow cytometry on mouse blood after NEFAs treatment on CD-1 mice was done on heparinized cardiac samples collected postmortem. The erythrocytes were lysed with FACS lysing solution as above. Here we used Alexa Fluor 488-AnnexinV (Thermo Fisher Scientific, MA, USA), and Alexa Fluor 700-CD3 (Biolegend, San Diego, CA, USA), eFluor450-GR-1 (Biolegend, San Diego, CA, USA), and PerCP/Cyanine5.5-CD45 (Biolegend, San Diego, CA, USA) antibodies were used for cell lineages marker. Cardiac blood of C57Bl6 mice after inducing pancreatitis with IL12&18 or IL12&18+GTL or IL12&18 +GTL+Orlistat was also collected in heparin tube. The heparinized blood (100µl) was mixed with citrate-phosphate-dextrose (CPD) buffer, and LIVE/DEAD Fixable Far-Red stain (1:1000 dilution, Thermo Fisher Scientific, MA, USA) in ice for 30 min with occasional gentle mixing. The blood was washed twice with CPD buffer. Then, erythrocytes were lysed like above. The WBCs were incubated with Alexa Fluor 488-CD68 (Biolegend, San Diego, CA, USA), APC-Cy7-GR-1 (Biolegend, San Diego, CA, USA), and PE-CD3 (Biolegend, San Diego, CA, USA). The excess antibodies were washed off. Unstained cell and respective isotype controls were used to check antibody specificity.   Labelled cells (10^5 counts/ tube) were studied on the BD LSRFortessa cytometer and analyzed using FlowJo Version 10 and BD FACSDiva™ Version 8.0.1.

Absolute CD3 number was calculated from the total WBC count multiplied by percentage of CD3 population in flow analysis for each mouse.

CELLULAR STUDIES:

Cell lines: Peritoneal macrophages were collected from CD1 wild type mouse. Briefly, 10-ml syringe was filled with 10 mL of cold harvest medium (PBS) and with the beveled end of a 20-G needle facing inward, the needle was inserted through peritoneal wall and all the 10 ml of the cold harvest medium was injected into each mouse. The mice were euthanized using CO2 and the peritoneal fluid was aspirated from the peritoneum using a 10mLsyringe and needle (24-G). The fluid recovery was ~8 ml per mouse. Further, the collected fluids were spun in at 1600g for 2 min to pellet down the peritoneal macrophages. The supernatant was removed, and cells were resuspended in PBS containing 0.1% albumin. These were then washed in pure PBS x3 to remove albumin and kept on ice before use. J774A.1 cells were maintained in Dulbecco's modified Eagle's medium (DMEM) (Corning, Manassas, VA) supplemented with 10% fetal bovine serum (Corning, Woodland, CA) and 1% penicillin/streptomycin (Life Technologies, Grand Island, NY). The cells were maintained in a humidified 5% CO_2

atmosphere at 37°C. The day before live imaging studies, the J774A.1 cells were seeded on glass bottom dish (MatTek, Ashland, MA) and incubated overnight at 37°C under 5% CO_2.

Mitochondrial depolarization in suspension: For in vitro mitochondrial depolarization studies, cells were loaded with (JC-1, 5 µg/ml: Enzo Life Sciences, Farmingdale, NY) at 37°C for 15 min in HEPES buffer (20 Mm HEPES, 120 mM NaCl, 5 mM KCl, 10 mM glucose, 10 mM sodium pyruvate) containing 0.1% albumin media. The cells were washed twice in HEPES buffer and after this stored on ice till just before use. Just before the experiment, the cells (e.g. peritoneal macrophage cell suspension (40 µl) was added to 1950 µL of PBS in a plastic fluorimeter cuvette equipped with a stir bar. Mitochondrial inner membrane potential values (Ψm) were determined by recording time-based fluorescence for excitation at 490 nm and alternate measuring emission at 530 and 590 nm using the temperature controlled F2710 Hitachi Fluorescence Spectrophotometer (excitation and emission slits 10.0 nm, Hz, stirring ~800 rpm). For e.g. for Figure 1K, after measurement for at least 100 s to ensure a stable signal, fatty acids (Oleic acid or Myristic acid or Lauric acid or Palmitoleic acid) were added at 2µM concentration to the above stirred cell suspensions through the injection port in the lid of the fluorimeter chamber, and fluorescence was further monitored for at least 500s. The data were collected every 10 s. The first 100s of data were averaged and subtracted, and the net increase was plotted as a function of time or FA concentrations. The 530/590 emission ratio for mito-depolarization were averaged and presented from at least five independent experiments**.**

Mitochondrial reactive oxygen species (MROS): cells were loaded with MitoSOX red using a protocol similar to JC1 mentioned above following the manufacturer's instructions (ThermoFisher). MROS were then measured in a stirred cuvette using excitation of 396nm and emission of 610nm in a manner similar to JC1. Mouse red blood cell lysate (final hemoglobin concentration of 0.5-0.7 mg/ml) with Hydrogen peroxide (1mM final concentration) were used as a positive control.

MitoTracker staining in adherent cells: MitoTracker Red CMXRos probes (Thermo Fisher Scientific, Eugene, OR) were dissolved in DMSO to make a 1mM stock. The stain was further diluted in a complete DMEM to a working concentration of 50nM. The adhering cells were stained for 20-30 minutes at 37°C, then washed twice with pre-warmed fresh HEPES buffer and studied with the ZEISS LSM 800 confocal microscope equipped with a 63x oil immersion lens and airyscan detector. Images were collected every 5 seconds before and after LA-Coumarin treatment.

FA-coumarin uptake into cells: Coumarin-fatty acids of Linoleic acid (LA; C18:2), oleic acid (OA; C18:1) and palmitic acid (PA; C16:2) with the coumarin group attached to the methyl terminus were custom-sythesized by Nanosyn Inc (Santa Clara, CA). Purity on LC-MS was confirmed to be >95%. These were dissolved in a chloroform to obtain a concentration of 2 mg/ml, aliquoted and stored at -87 °C until use. On the day of the assay, 7.5µl of chloroform solution of FA-Coumarin was placed into a glass vial and dried under nitrogen. The dry FA-Coumarin was then resuspended in 1µl 3.2M corresponded FA and 320µl warm PBS and sonicated to make a mixture of 10mM FA + 167µM FA-Coumarin. A reconstituted FA-Coumarin suspension was diluted in a warm HEPES buffer to get a proper concentration, sonicated after each dilution, and used for cell treatment at 37°C. In adherent live cell monolayers LA-coumarin was reproducibly visualized at ≈1µM (total LA > 30 µM; Supplementary figure 6).This was necessary to prevent photobleaching, allow reliable visualization despite uptake into cells via only one cell surface exposed to the NEFA. Additionally, avoidance of stirring was necessary for phagocytosis studies described below. Moreover, imaging of single cell monolayers can inherently reduce sensitivity compared to the several cells (100s or more) in the light path of a stirred cell suspension in a cuvette.

Phagocytosis assays: pHrodo™ Red E. coli BioParticles™ Conjugate (pH dependent dye conjugated with E. coli, (Thermo Fisher Scientific, Eugene, OR) were resuspended and sonicated in HEPES buffer to make 1 mg/ml stock. On the day of the assay, adhering J774A.1 cells were stained with 50nM LysoTracker Green DND-26 (Thermo Fisher Scientific, Eugene, OR) for 20-30 minutes at 37°C, then washed twice with pre-warmed fresh HEPES. BioParticles were added to cells at 1:15 for a final concentration of 70µg/ml after LA-Coumarin treatment. Live imaging was performed for 90 min, with 2-minutes intervals between shots. Phagocytosis was quantified by measuring the fluorescence intensity of colocalization between LysoTracker and pHrodo™ Red E. coli using Fiji (ImageJ) software.

ATP levels on live imaging: To visualize ATP levels inside individual living cells we used FRET-based ATeam sensor consisting of the ε-subunit of the bacterial F_0F_1-ATP synthase sandwiched by CFP and YFP

(Addgene, cat#: 51958). J774A.1 cells were transiently transfected with ATeam1.03-nD/nA/pcDNA3 construct using Xfect Transfection Reagent (TaKaRa, cat#: 631317). Live cell images were taken using confocal microscope equipped with a 405-nm diode laser every 30 seconds before and after LA/OA treatment. The band-pass emission filters were used as follows: 455-550 nm (CFP) and 507-586 nm (YFP). Quantification and processing including background subtraction, were done in Fiji (ImageJ) software. Ratios of CFP/YFP were used to measure ATP levels.

Bioluminescent Measurement of ATP: A bioluminescent-based assay kit was used to measure intracellular ATP levels (CellTiter-Glo® 2.0 Assay, Promega, Madison, USA) following manufacturer's instructions in cells (Fig 3D). Briefly, J774A.1 and peritoneal macrophages were suspended in HEPES buffer (with 0.01% albumin for e.g. in Fig 3D) in appropriate dilution and added to white 96 well plate (Greiner bio-one, Germany) and exposed to respective fatty acids at unbound concentrations calculated based on the calibration curve in supplemental figure 7. and incubated at 37°C with shaking at 120rpm for designated time points. At the end point, equal volume of assay buffer containing luminescent substrate was added to each well and measured on a Promega Glomax 20/20 Luminometer. Percentage of ATP coming from the viable cells for a particular dose and time was calculated considering the control set ATP level as 100%.
Biochemical assays: Lactate dehydrogenase (LDH) was measured in the medium of cells before and after treatment. The increase in leakage was normalized to total LDH content in the medium after cell lysis using 1% tritone for 1 hour or more.

Voltage dependent anion channel (VDAC) oligomerization studies: The J774A.1 cells were cultured in 10-cm plates in DMEM supplemented with 10% FBS in a humidified atmosphere at 37 °C with 5% CO2. When cells reached 80% confluency, the medium was replaced with HEPES buffer (3 washes) and cells were treated with different concentrations of fatty acids within different time frames. To reverse fatty acid induced VDAC oligomerization, cells were treated with 1% albumin for 5 min at 37 °C. The cells were then incubated with cross-linking reagent (Ethylene glycol-bis(succinic acid N-hydroxysuccinimide ester), EGS) (Sigma-Aldrich, St. Louis, MO) for 15 min at 37 °C. After that, the EGS reagent was discarded, Following that, cells were harvested with a lysis buffer composed of Native PAGE Sample Buffer (Life Technologies, Carlsbad, CA), Digitonin (1.25% final concentration) (Life Technologies, Carlsbad, CA) and Benzonase Nuclease (2 units/μl final) (Sigma-Aldrich, St. Louis, MO), incubated at room temperature for 40 min and centrifuged at 20,000 x g for 30 min at 4 °C. The supernatant was aliquoted into sterile microcentrifuge tubes and stored at -87 °C until use. Protein concentration was determined using Pierce BCA Protein Assay Kit (Thermo Scientific, Rockford, IL). Then, G-250 Sample Additive (0.32% final concentration) (Life Technologies, Carlsbad, CA) was added to the samples. For Western blotting, sample (10 to 20 μg) were separated by electrophoresis using SDS-PAGE gels. Gels were electrotransferred onto Immobilon-P membranes (Millipore, Carrigtwohill, Ireland). The blots were blocked in a blocking solution containing 5% non-fat dry milk and 0.1% Tween-20 in Tris-buffered saline, followed by incubation with anti-VDAC1/2 (1:1000) antibodies (Abcam, ab154856).

LIPIDOMICS STUDIES:

Unbound fatty acid characterization by gas chromatography and mass spectrometry (GC-MS) : These were previously detailed by Cartin-Ceba et al[15]. Individual steps in sequence were albumin depletion using the Pierce Albumin Depletion Kit (Rockford, IL), followed by lipid extraction, derivatization using Deoxo-Fluor method by Kangani et al[16]. Derivatized fatty acids were quantified using GC-MS on the Agilent GC 7890B system attached to an Agilent 5977A MSD, with a HP-5MS (UI 30 m x .25mm I.D. with a .25μm fil thickness) capillary column. The internal standard added to every sample at the time of extraction to account for extraction efficiency comprised of 4.5μM C16:2, 9.1μM C17:0, and 9.1μM C19:2. A 5ppm caffeine in hexane mixture was used for injection to determine the GC consistency throughout a run. A standard curve was made in heptane and serial diluted from 20μM down to 0.1μM comprised of C12:0, C14:0, C16:0, C16:1, C18:0, C18:1, C18:2, C18:3 and C20:4 which were all quantified for each sample. Blanks of hexane were taken through the extraction process and subtracted out of the GC response for each sample and standard. The individual fatty acid values for each sample are then calculated based on the linear equation based on the standard curve. Values were reported a micromolars after being adjusted based on the internal standards, and accounting for extraction efficiency.

Serum non esterified fatty acid assay by GC: The serum samples frozen at -80C were transported on dry ice to the Vanderbilt university medical center lipidomics core, and analyzed for individual NEFA composition using GC as described previously[11, 14, 15]. The result were reported as micromolars.

Thin layer chromatography (TLC) of J774A.1 lipids for LA uptake, reversal by albumin: J774A.1 cells were grown in 10cm plate and once they reach 80-90 % confluency cells were recovered by trypsin digestion and collected in 1.5ml microfuge tube. Linoleic acid stock (3.2M) was initially sonicated (three pulses - 10 sec ON, 5 sec OFF) into the HEPES buffer (pH 7.4) (20 Mm HEPES, 120 mM NaCl, 5 mM KCl, 10 mM glucose, 10 mM sodium pyruvate). This was further diluted to working stock of 10mM linoleic acid concentration by immediately diluting in HEPES buffer followed by sonication as previously described. The working stock prepared was either promptly used or else was incubated at 37°C till the time of use. This stock was used to get the final concentration of 50µM linoleic acid in the cell suspension.

Cells from each 10cm plate were either treated with 50µM Linoleic acid (LA) alone for 2 min or with LA (50µM) followed by albumin (0.5%) after 2 min of LA addition or untreated (control). The cells were washed once with PBS and further proceeded with lipid extraction. Total lipids were extracted by the Folch method and the lipids were recovered in the chloroform phase and further separated by thin-layer chromatography using Silica Gel G plates (Analtech, Newark, DE). Standards were loaded next to the samples to identify the lipids. Lipids were visualized by spraying with primulin (25mg of primulin in 10mL of water and 40mL of acetone) followed by viewing on UV transilluminator.

At the time of TLC, the TLC tank was filled with 100mL of a 1:1 methanol chloroform mixture. A filter paper was placed along the wall of the tank and allowed it to saturate. Once the filter paper had saturated, the Silica Gel G plate was placed on the opposite wall facing towards the filter paper. Once the solvent front migrated to the top of the plate, the plate was taken out and allowed to air dry in a fume hood for 5-10 minutes before moving it to a 60°C dry incubator for a minimum of 4 hours. Prepare a running solvent of 60mL chloroform, 70mL of ethanol, 14 mL of D.I. water and 70mL of triethylamine and add that to the TLC tank. Place the filter paper on the wall and allow to saturate again. Reactivate the silica on the dry TLC plate by placing it onto a hot plate set to 130°C for 3 minutes. Spot the standards and samples on the plate and then allow the plate to dry for 5-10 minutes after spotting. The plate was added to the opposite wall of the filter paper in the tank like before and allow the solvent to migrate until the solvent front is about 1 inch from the top of the plate. Then, the plate was removed from the tank and allowed to air dry for 5 minutes before spraying the plate with the primulin solution. After the spray dried up, the plate was viewed under UV light.

Liposome Fatty acid interaction/binding:

We used a modified method, adapted from Manley and Gordon (2008) using dehydration and rehydration of large unilamellar vesicles (LUVs) to produce giant unilamellar vesicles (GUVs)[17]. The protocol for LUV preparation is a variation of a previously established method[18].

Briefly, 4.2 µmol of total lipids containing a mixture of POPC, and Cy5-DOPC (0.5 mol %) was dried in a glass tube under gentle nitrogen stream. This was further incubated in a vacuum desiccator for a minimum of 3 hours to overnight. After incubation, the lipid film in the vial was hydrated with 1 mL of 100 mM NaCl containing 1 vol% of glycerol. This was subjected to sonication in a bath sonicator for 2 minutes to dissociate the lipid film from the glass bottom and make a uniform lipid suspension. Further, this suspension was given three cycles of freeze–thaw (freezing in liquid nitrogen and thawing in a 37 °C water bath) and was further extruded in an Avanti Mini Lipid Extruder by giving 21 passes through 400nm trac-ketch membrane followed by 15 passes through 200-nm track-etch membrane to generate the 200nm LUVs. These LUVs were kept at 4 °C and were used within 2 days. From the above LUV suspension, 10 µL was spread onto a glass cover slip. This was allowed to dry for 30 min and further rehydrated with 50 µl of buffer (50 mM HEPES–NaOH, pH 7.4, 100 mM NaCl). After around 5 minutes, giant unilamellar vesicles (GUVs) start to form which are then collected and visualized. Since the lipid mixture contained light-sensitive Cy5-DOPC, liposomes (LUVs or GUVs) were protected from light throughout the experiment. For fatty acid (Oleic acid) binding experiment with GUVs, sonicated oleic acid stocks solutions containing Top Fluor OA as tracer were freshly prepared and kept at 37 ºC during the experiment. Liposomes were mixed with Oleic acid stock solution on slides just before imaging to get a final concentration of 150µM of OA and 2.0µM of Top Fluor OA. These were imaged at 37ºC under a 63X oil immersion objective in a Zeiss LSM 800 confocal microscope (Zeiss, Oberkochen, Germany).

Statistics: Data depicted from *in vitro* assays is from at least five independent experiments, while animal studies included 8-12 animals in each group. The rationale for number of human patients included in each group was determined by the recruitment over the study period and is detailed in the first part of the results section. Categorical data in two groups were compared using the Fisher-Exact test. Continuous data in two groups were compared using a Student's T-test, or Mann-Whitney test, depending on the normality of distribution of samples. Multiple groups were compared by ANOVA for samples with a normal distribution or a Kruskal–Wallis H test (one-way ANOVA on ranks) where the distribution was skewed. A P value of < 0.05 was regarded as significant (*). Each additional * depicts a decrease in P by 1/10. Groups are compared using box plots with median line, and error bars depicting range. Line graphs connect means, and error bars depict standard error of mean.

SUPPLEMENTARY REFERENCES:

[1] Klindworth A, Pruesse E, Schweer T, Peplies J, Quast C, Horn M, Glockner FO: Evaluation of general 16S ribosomal RNA gene PCR primers for classical and next-generation sequencing-based diversity studies. Nucleic Acids Res 2013, 41:e1.
[2] Katoh K, Misawa K, Kuma K, Miyata T: MAFFT: a novel method for rapid multiple sequence alignment based on fast Fourier transform. Nucleic Acids Res 2002, 30:3059-66.
[3] Bolyen E, Rideout JR, Dillon MR, Bokulich NA, Abnet CC, Al-Ghalith GA, Alexander H, Alm EJ, Arumugam M, Asnicar F, Bai Y, Bisanz JE, Bittinger K, Brejnrod A, Brislawn CJ, Brown CT, Callahan BJ, Caraballo-Rodríguez AM, Chase J, Cope EK, Da Silva R, Diener C, Dorrestein PC, Douglas GM, Durall DM, Duvallet C, Edwardson CF, Ernst M, Estaki M, Fouquier J, Gauglitz JM, Gibbons SM, Gibson DL, Gonzalez A, Gorlick K, Guo J, Hillmann B, Holmes S, Holste H, Huttenhower C, Huttley GA, Janssen S, Jarmusch AK, Jiang L, Kaehler BD, Kang KB, Keefe CR, Keim P, Kelley ST, Knights D, Koester I, Kosciolek T, Kreps J, Langille MGI, Lee J, Ley R, Liu YX, Loftfield E, Lozupone C, Maher M, Marotz C, Martin BD, McDonald D, McIver LJ, Melnik AV, Metcalf JL, Morgan SC, Morton JT, Naimey AT, Navas-Molina JA, Nothias LF, Orchanian SB, Pearson T, Peoples SL, Petras D, Preuss ML, Pruesse E, Rasmussen LB, Rivers A, Robeson MS, 2nd, Rosenthal P, Segata N, Shaffer M, Shiffer A, Sinha R, Song SJ, Spear JR, Swafford AD, Thompson LR, Torres PJ, Trinh P, Tripathi A, Turnbaugh PJ, Ul-Hasan S, van der Hooft JJJ, Vargas F, Vázquez-Baeza Y, Vogtmann E, von Hippel M, Walters W, Wan Y, Wang M, Warren J, Weber KC, Williamson CHD, Willis AD, Xu ZZ, Zaneveld JR, Zhang Y, Zhu Q, Knight R, Caporaso JG: Reproducible, interactive, scalable and extensible microbiome data science using QIIME 2. Nat Biotechnol 2019, 37:852-7.
[4] Callahan BJ, McMurdie PJ, Rosen MJ, Han AW, Johnson AJ, Holmes SP: DADA2: High-resolution sample inference from Illumina amplicon data. Nat Methods 2016, 13:581-3.
[5] Price MN, Dehal PS, Arkin AP: FastTree 2--approximately maximum-likelihood trees for large alignments. PLoS One 2010, 5:e9490.
[6] Faith DP: Conservation evaluation and phylogenetic diversity. Biological Conservation 1992, 61:1-10.
[7] Lozupone CA, Hamady M, Kelley ST, Knight R: Quantitative and qualitative beta diversity measures lead to different insights into factors that structure microbial communities. Appl Environ Microbiol 2007, 73:1576-85.
[8] Lozupone C, Knight R: UniFrac: a new phylogenetic method for comparing microbial communities. Appl Environ Microbiol 2005, 71:8228-35.
[9] Schierwagen R, Alvarez-Silva C, Servant F, Trebicka J, Lelouvier B, Arumugam M: Trust is good, control is better: technical considerations in blood microbiome analysis. Gut 2020, 69:1362-3.
[10] Lu JD, Cao F, Ding YX, Wu YD, Guo YL, Li F: Timing, distribution, and microbiology of infectious complications after necrotizing pancreatitis. World J Gastroenterol 2019, 25:5162-73.
[11] Khatua B, El-Kurdi B, Patel K, Rood C, Noel P, Crowell M, Yaron JR, Kostenko S, Guerra A, Faigel DO, Lowe M, Singh VP: Adipose saturation reduces lipotoxic systemic inflammation and explains the obesity paradox. Sci Adv 2021, 7.
[12] Noel P, Patel K, Durgampudi C, Trivedi RN, de Oliveira C, Crowell MD, Pannala R, Lee K, Brand R, Chennat J, Slivka A, Papachristou GI, Khalid A, Whitcomb DC, DeLany JP, Cline RA, Acharya C, Jaligama D, Murad FM, Yadav D, Navina S, Singh VP: Peripancreatic fat necrosis worsens acute pancreatitis independent of pancreatic necrosis via unsaturated fatty acids increased in human pancreatic necrosis collections. Gut 2016, 65:100-11.

[13] Navina S, Acharya C, DeLany JP, Orlichenko LS, Baty CJ, Shiva SS, Durgampudi C, Karlsson JM, Lee K, Bae KT, Furlan A, Behari J, Liu S, McHale T, Nichols L, Papachristou GI, Yadav D, Singh VP: Lipotoxicity causes multisystem organ failure and exacerbates acute pancreatitis in obesity. Sci Transl Med 2011, 3:107ra10.
[14] de Oliveira C, Khatua B, Noel P, Kostenko S, Bag A, Balakrishnan B, Patel KS, Guerra AA, Martinez MN, Trivedi S, McCullough A, Lam-Himlin DM, Navina S, Faigel DO, Fukami N, Pannala R, Phillips AE, Papachristou GI, Kershaw EE, Lowe ME, Singh VP: Pancreatic triglyceride lipase mediates lipotoxic systemic inflammation. J Clin Invest 2020, 130:1931-47.
[15] Cartin-Ceba R, Khatua B, El-Kurdi B, Trivedi S, Kostenko S, Imam Z, Smith R, Snozek C, Navina S, Sharma V, McFayden B, Ionescu F, Stolow E, Keiser S, Tejani A, Harrington A, Acosta P, Kuwelker S, Echavarria J, Nair GB, Bataineh A, Singh VP: Evidence Showing Lipotoxicity Worsens Outcomes in Covid-19 Patients and Insights About the Underlying Mechanisms. iScience 2022:104322.
[16] Kangani CO, Kelley DE, Delany JP: New method for GC/FID and GC-C-IRMS analysis of plasma free fatty acid concentration and isotopic enrichment. J Chromatogr B Analyt Technol Biomed Life Sci 2008, 873:95-101.
[17] Manley S, Gordon VD: Making Giant Unilamellar Vesicles via Hydration of a Lipid Film. Current Protocols in Cell Biology 2008, 40:24.3.1-.3.13.
[18] Khelashvili G, Pillai AN, Lee J, Pandey K, Payne AM, Siegel Z, Cuendet MA, Lewis TR, Arshavsky VY, Broichhagen J, Levitz J, Menon AK: Unusual mode of dimerization of retinitis pigmentosa-associated F220C rhodopsin. Scientific Reports 2021, 11:10536.

Supplementary Figures:

Supplementary Figure 1: Boxplots showing total number of 16S DNA copies amplified at 35 cycles, that were analyzed for each sample among different groups: There was no significant difference between the groups.

Supplementary Figure 2: Linear discriminant analysis (LDA) effect size (LEFSe) comparison of human serum microbiome data: Human data of AP patients (green) and controls (red) comparing genera enriched on a Log 10 LDA score of > 3.6 in AP patients. These included Pseudomonadales (please see Fig 1H) *Acinetobacter* (top row in green) and *Pseudomonas* (third from top row).

Supplementary Figure 3: Level 4 Taxa-bar plots of human serum microbiome amplified at 35 cycles showing the increasing proportion of "unassigned DNA" (grey) and Pseudomonadales (pink) in patients with pancreatitis, pancreatitis with infection.

Supplementary Figure 4: Flowcytometry on human samples: Data in each column and row represents the title (bold text) mentioned above the column and to the left of the row respectively. Scattergrams in the second, 3rd rows show annexin V (Alexa Fluor 488) staining on the Y-axis and CD3, CD14 staining on the X-axis respectively. The histograms on the top compare annexin V staining in total population of cells. On the extreme right compare the Annexin V positive populations in the healthy controls (orange), non-infected patients (blue) and infected patients (red).

Supplementary Figure 5: Tables comparing the groups of decedents whose autopsy samples were analyzed: A: Table of demographics, etiology of AP, and isolated organism in patients with documented infected necrosis (top rows) and those with no infection documented (bottom rows). **B:** Table describing characteristics of controls **C:** Table comparing Age, sex, BMI of AP patients with controls.

Supplementary Figure 6: Graphs describing the sensitivity of the live fluorescence imaging system: A: Time courses of mitochondrial depolarization induced by different concentrations of LA (corresponding colors shown to right), with 2% LA-coumarin uptake into cells shown in **B**. The fatty acids (FA) were added at 60 seconds, and albumin was added at 210 seconds.

Supplementary Figure 7: Calibration curve for unbound fatty acid concentrations present in 0.01% albumin in PBS. X-axis indicates the amount of oleic acid in the medium, and Y-axis shows the unbound fatty acid concentration measured using the kit from FFA sciences as described in methods.

Supplementary Figure 8: Flowcytometry of blood of control mice and 4-5 hours after LA, or LA + albumin treatment stained for GR-1 and annexin V: Each treatment group is shown in a different row. In these scattergrams X-axis shows staining for GR-1 and Y-axis shows staining for Annexin V from 2 mice in each group.

Supplementary Figure 9: Flowcytometry of blood of control mice and 4-5 hours after LA, or LA + albumin treatment stained for CD3 and annexin V: Each treatment group is shown in a different row. In these scattergrams X-axis shows staining for CD3 and Y-axis shows staining for Annexin V from 2 mice in each group.

Supplementary Figure 10: Scatterplots of day 3 carotid artery pulse distention (A) and serum BUN at the time of necropsy (B) in rats from different groups with pancreatitis who had received GFP *E. coli* via gavage. the P value shown represents ANOVA.

Supplementary Figure 11: Two examples each of flowcytometry on blood from control mice, those with IL12,18 pancreatitis, modified by GTL ± orlistat stained for lineage markers (X-axis) and live dead marker (Y-axis): Lineage markers are CD68 (left panel), GR-1 (middle panel), and CD11b (right panel). Each pair in a row represents samples from the treatment group mentioned on the extreme left.

Supplementary Figure 12: Histograms of flowcytometry on blood from control mice, those with IL12,18 pancreatitis, modified by GTL ± orlistat stained for live dead marker in over all white cell population: Each row represents samples from the treatment group mentioned on the extreme left. The table on the right shows the percentage dead cells for each sample arranged by treatment groups.

Supplementary Figure 13: Boxplots of serum cytokines (TNF-α, IL-10, IL-6, MCP-1 and IL-1β) for each group of animal experiments in the order these appear in the study. The boxes represent inter-quartile range and the error bars the sample range. P values shown above compare ANOVAs to controls. The serums were collected at the time of necropsy.

<u>Supplementary Videos:</u>

Supplementary Video 1. J774A.1 cells were loaded with MitoTracker (Red) and treated with LA-Coumarin (white) after 25 seconds of starting the video. The serial images were taken every 5 seconds.

Supplementary Video 2. J774A.1 cells were loaded with JC-1, where red is the baseline polymeric state and green is the monomeric state during mitochondrial depolarization. Cells were treated with LA-Coumarin (white) after 30 seconds baseline. Albumin with Alexa 647 tracer (purple) was added after 4 minutes recording. The serial images were taken every 30 seconds.

Supplementary Video 3. J774A.1 cells were loaded with JC-1, where red is the baseline polymeric state and green is the monomeric state during mitochondrial depolarization. Cells were treated with OA-Coumarin (white) after 30 seconds baseline. Albumin with Alexa 647 tracer (purple) was added after 4 minutes recording. The serial images were taken every 30 seconds.

Supplementary Video 4. J774A.1 cells were loaded with JC-1, where red is the baseline polymeric state and green is the monomeric state during mitochondrial depolarization. Cells were treated with PA-Coumarin (white) after 30 seconds baseline. Albumin with Alexa 647 tracer (purple) was added after 4 minutes recording. The serial images were taken every 30 seconds.

Supplementary Video 5. J774A.1 cells were transfected with FRET-based ATeam sensor. A high level of YFP fluorescence is associated with a high ATP level, whereas increasing CFP fluorescence corresponds to a reduced level of ATP. Cells were treated with LA-Coumarin (white) after 1 minute baseline. Albumin with Alexa 647 tracer (purple) was added after 4 minutes recording. The serial images were taken every 30 seconds.

Supplementary Video 6. J774A.1 cells were transfected with FRET-based ATeam sensor. A high level of YFP fluorescence is associated with a high ATP level, whereas increasing CFP fluorescence corresponds to a reduced level of ATP. Cells were treated with OA-Coumarin (white) after 1 minute baseline. Albumin with Alexa 647 tracer (purple) was added after 4 minutes recording. The serial images were taken every 30 seconds.

Supplementary Video 7. J774A.1 cells were stained with LysoTracker (green). pHrodo Red E. coli were visualized after 8 min baseline. The serial images were taken every 2 minutes.

Supplementary Video 8. J774A.1 cells were stained with LysoTracker (green). Cells were imaged prior to addition of LA-Coumarin (First image) and after treatment with LA-Coumarin snapshots were taken at every 2 min interval. pHrodo Red E. coli were visualized after 18 min of LA-Coumarin addition. The serial images were taken every 2 minutes.

Supplementary Video 9. J774A.1 cells were stained with LysoTracker (green). Cells were imaged prior to addition of LA-Coumarin (First image) and after treatment with LA-Coumarin snapshots were taken at every 2 min interval. pHrodo Red E. coli were visualized after 5 min, and LA-Coumarin was added at 10minutes. Albumin with Alexa 647 tracer (purple) was added after 20 minutes of recording.
