# Supplementary material for: Amphipathic Liponecrosis Impairs Bacterial Clearance and Causes Infection During Sterile Inflammation
Source: Gastroenterology. Author manuscript; Available in PMC 2023 Dec 7. (PMC10703425; doi:10.1053/j.gastro.2023.05.034)
Supplement: 2 [file NIHMS1905536-supplement-2.pdf]

Supplemental figure 1

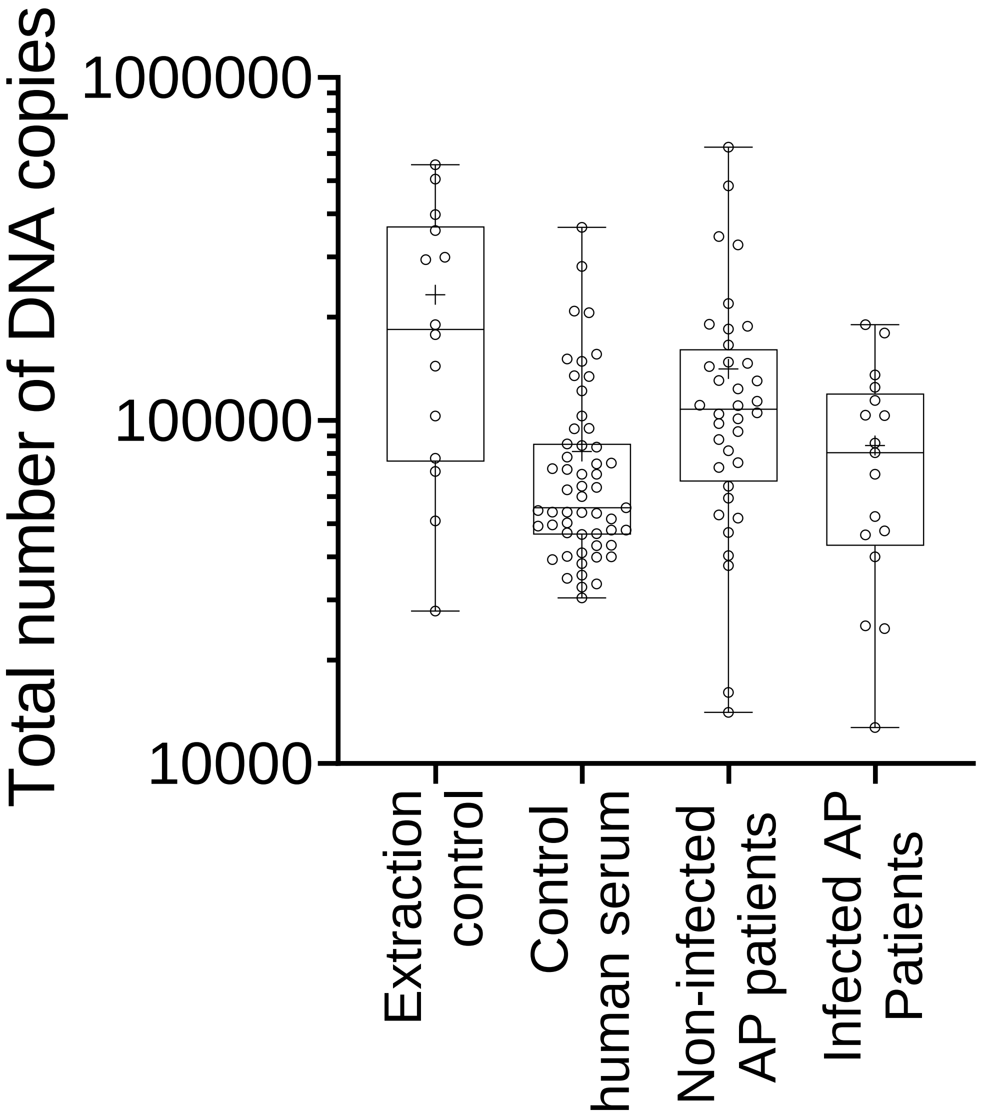

Supplemental figure 2

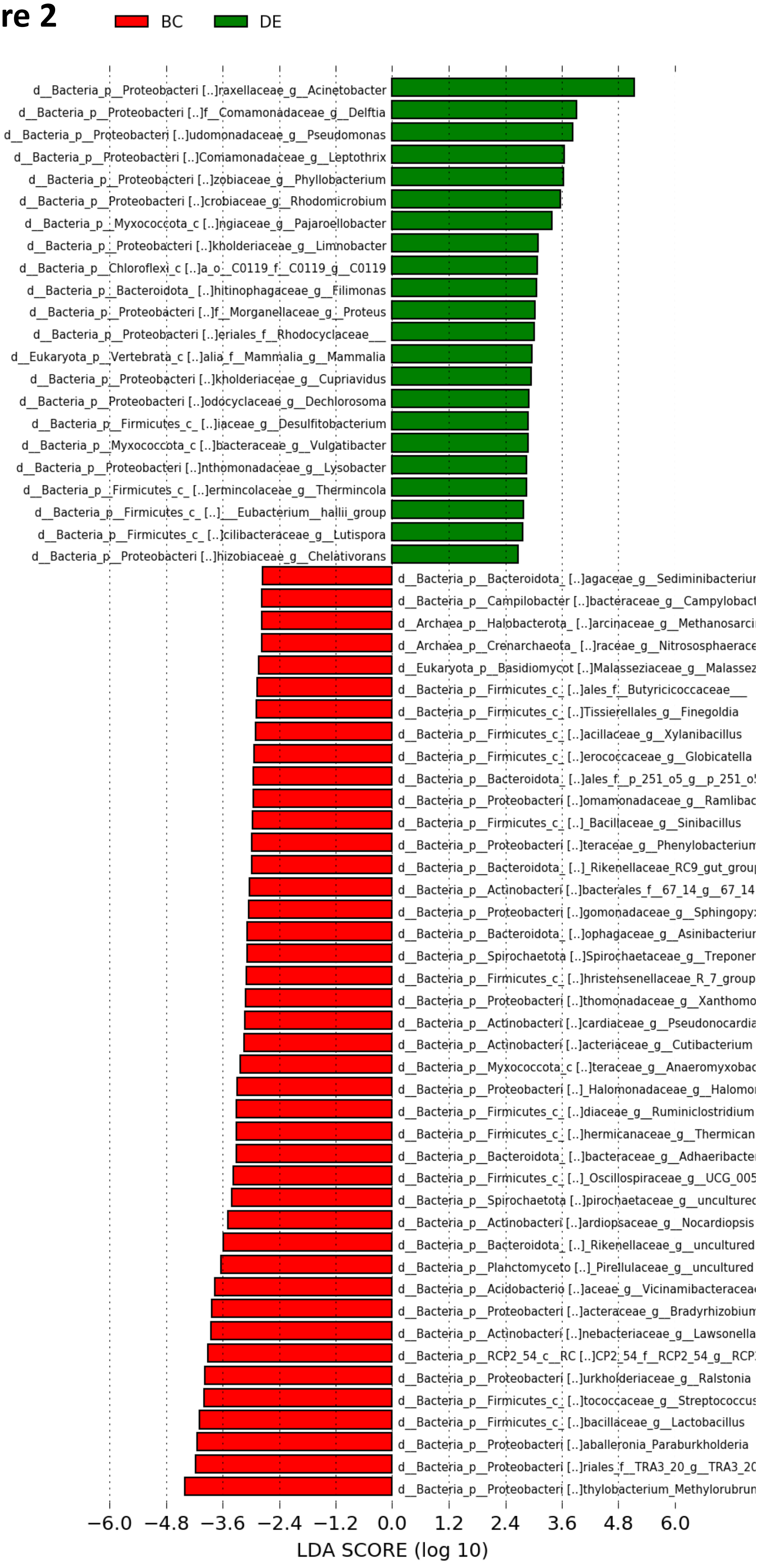

Supplemental figure 3

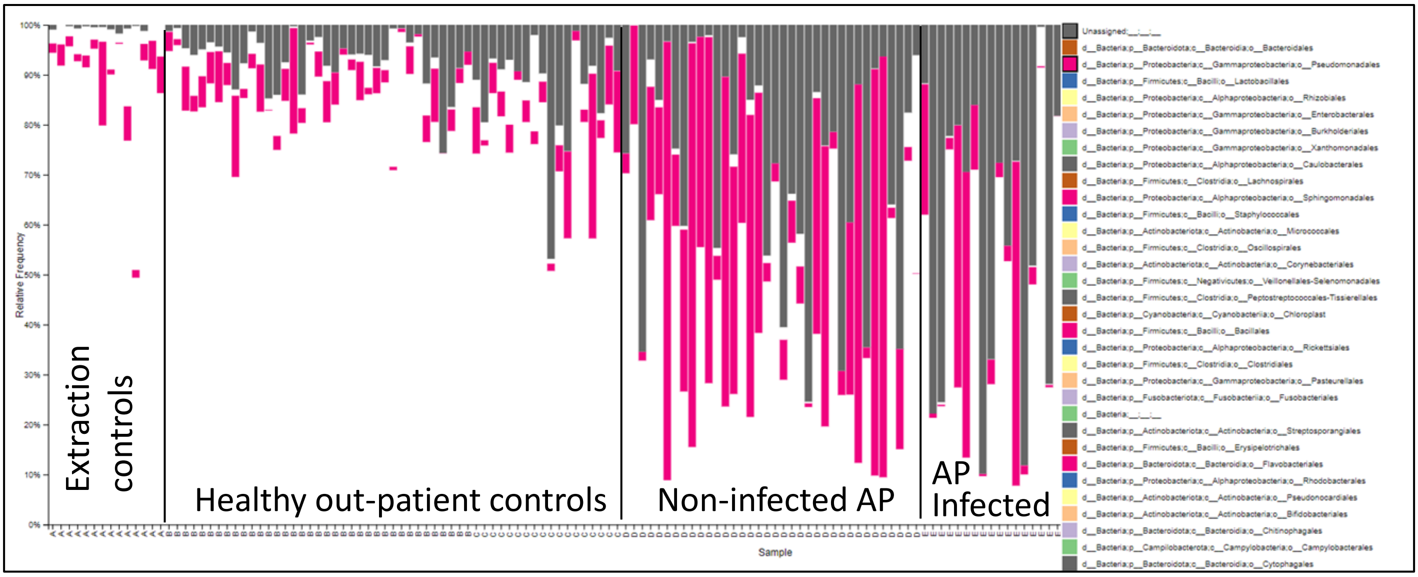

Supplemental figure 4

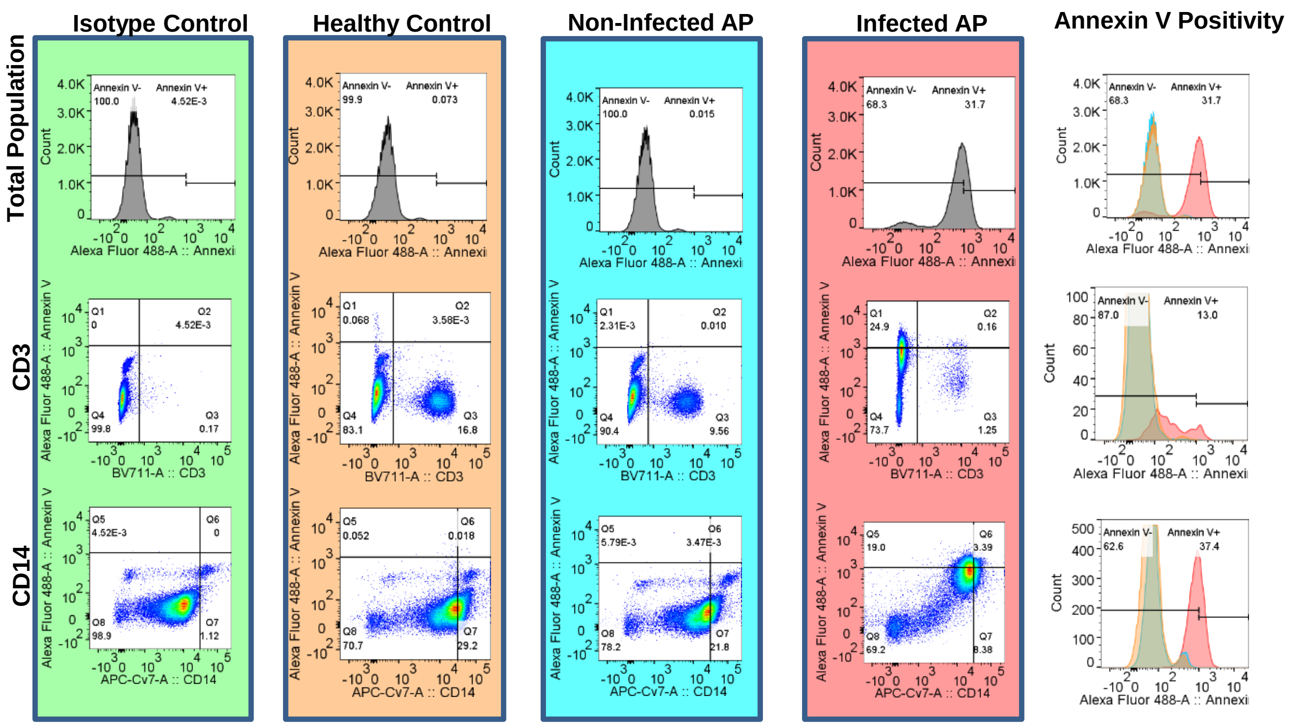

Supplemental figure 5

Clinical feature of decedents undergoing autopsy

A Clinical features of necrotizing AP group

	Pt. No.	Age	Sex	BMI	Etiology of Pancreatitis	Debridement Cult. Result
Documented infected necrosis	1	28	M	24.0	gallstone	E Coli, VRE
	2	45	F	NA	Gallstone	Yeast, Enterococcus, Klebsiella
	3	60	M	NA	Gallstone	Candida
	4	75	F	28.3	idiopathic	E. coli, Yeast
No infection documented antemortem	5	66	M	30.5	hyperlipidemia	Enterococcus
	6	32	M	19.9	Gallstone	Staph. aureus
	7	54	M	39.0	post ERCP	NA
	8	48	M	56.1	Gallstones	NA
	9	72	M	35.2	Gallstone	NA
	10	42	M	33.4	idiopathic	NA

B Clinical features of control group

Pt. No.	Age	Sex	BMI	cause of death	Primary diagnosis
1	75	M	22.6	Hypoxic resp. failure	Transverse myelitis
2	74	F	NA	shock	Metastatic carcinoma of the right breast
3	52	F	16.9	GI bleed	CHF
4	63	F	34.9	hypoxic resp. failure	PBC (post liver transplant)
5	70	M	26.1	Cardiorespiratory Failure	Usual Interstitial Pneumonia
6	77	M	21.6	Cardiorespiratory Failure	Pulm. HTN
7	53	M	34.2	Cardiogenic shock (Acute MI)	Valvular cardiomyopathy with CHF
8	63	M	20.7	Hypoxic resp. failure	multifocal hemorrhagic pneumonia
9	54	M	31.6	Necrotizing bronchopneumonia	Staph aureus pneumonia

C Comparison of Control and AP groups

	Control	Necrotiz. AP	*P*-value
**Age**	64.6 ± 9.9	52.2 ± 16.1	0.06
**M:F**	6:3	8:2	0.67
**BMI**	26.1 ± 6.8	33.2 ± 11.0	0.14
**LOS(Days)**	22.2 ± 20	8.6 ± 6	0.062

Supplemental figure 6

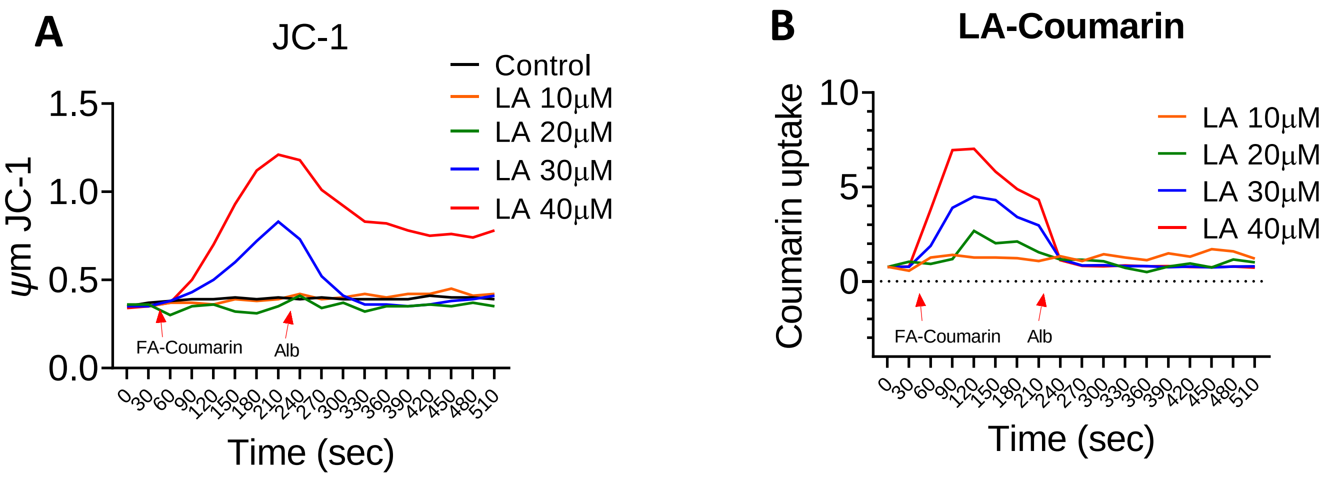

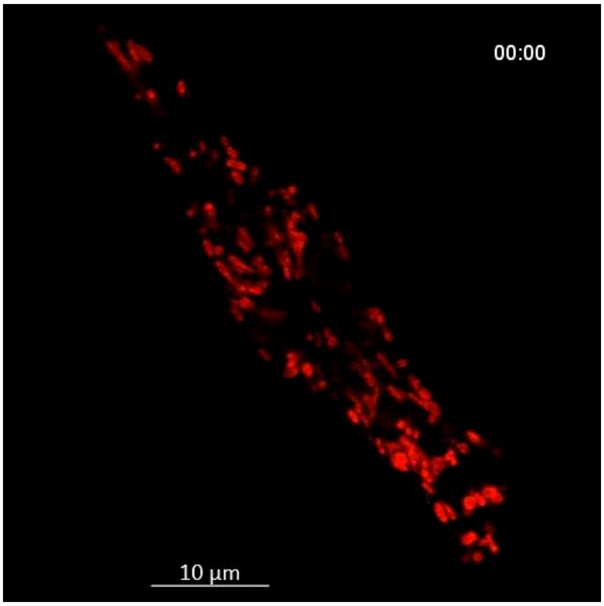

Supplementary movie-1 – LA-coumarin uptake in mitotracker loaded J774A.1 cells images every 5 seconds

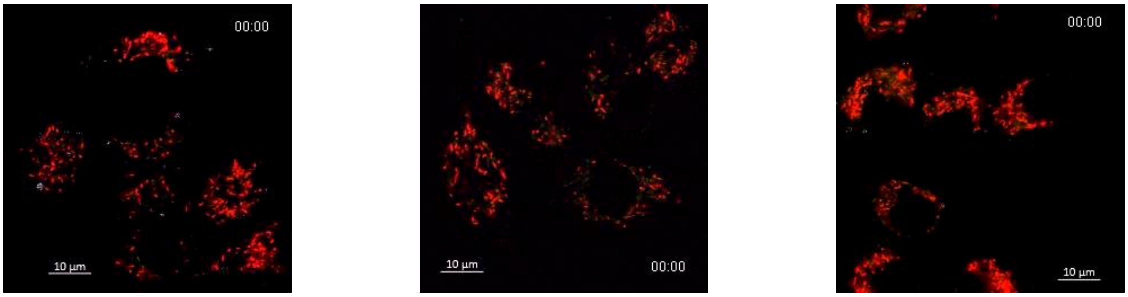

Supplementary movie-2-4 – LA-coumarin vs. OA-coumarin vs. PA-coumarin uptake in J774A.1 cells loaded with JC-1

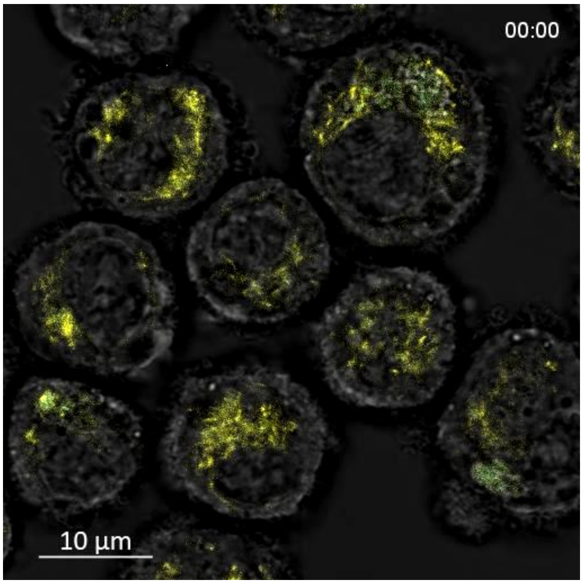

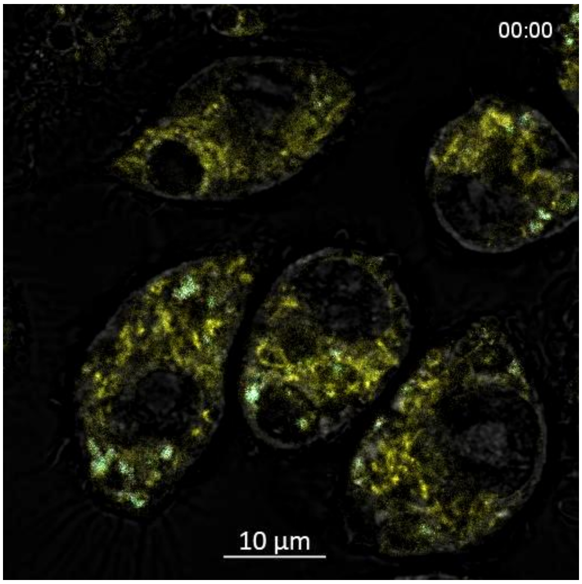

Supplementary movie-5, 6 – ATP biosensor data for LA, OA,

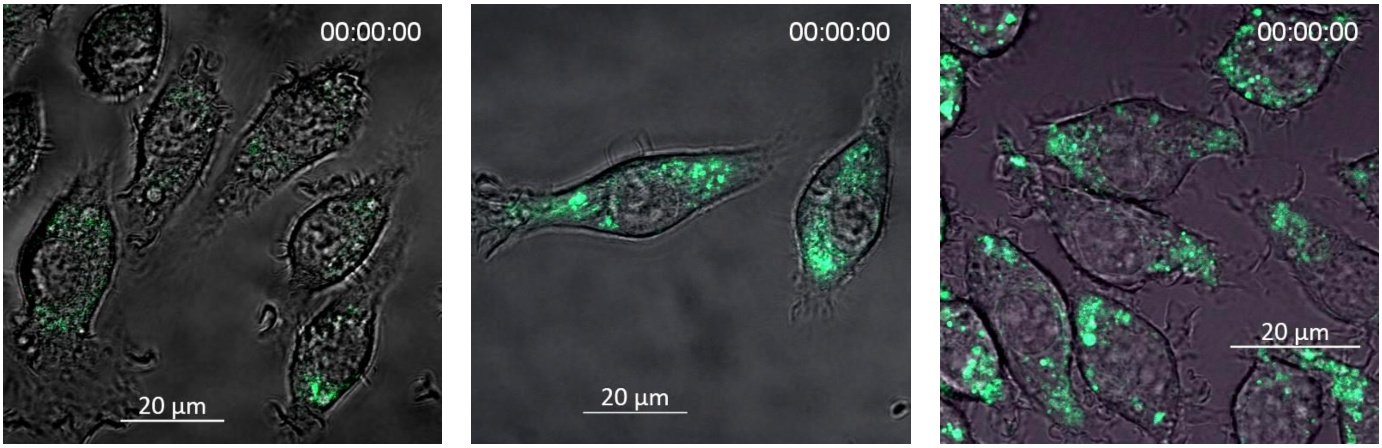

Supplementary movie-7, 8, 9 – E.coli phagocytosis by control (7) and LA treated cells (8) and normalization by albumin (9)

Supplemental figure 7

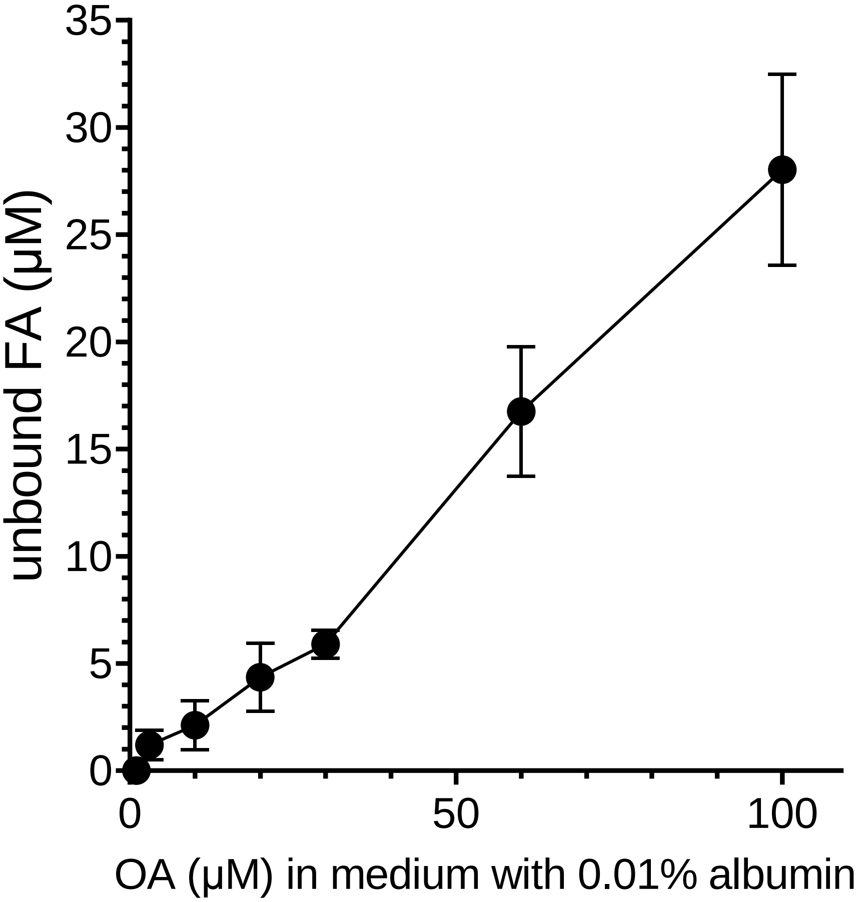

Supplemental figure 8

GR-1 positive cells

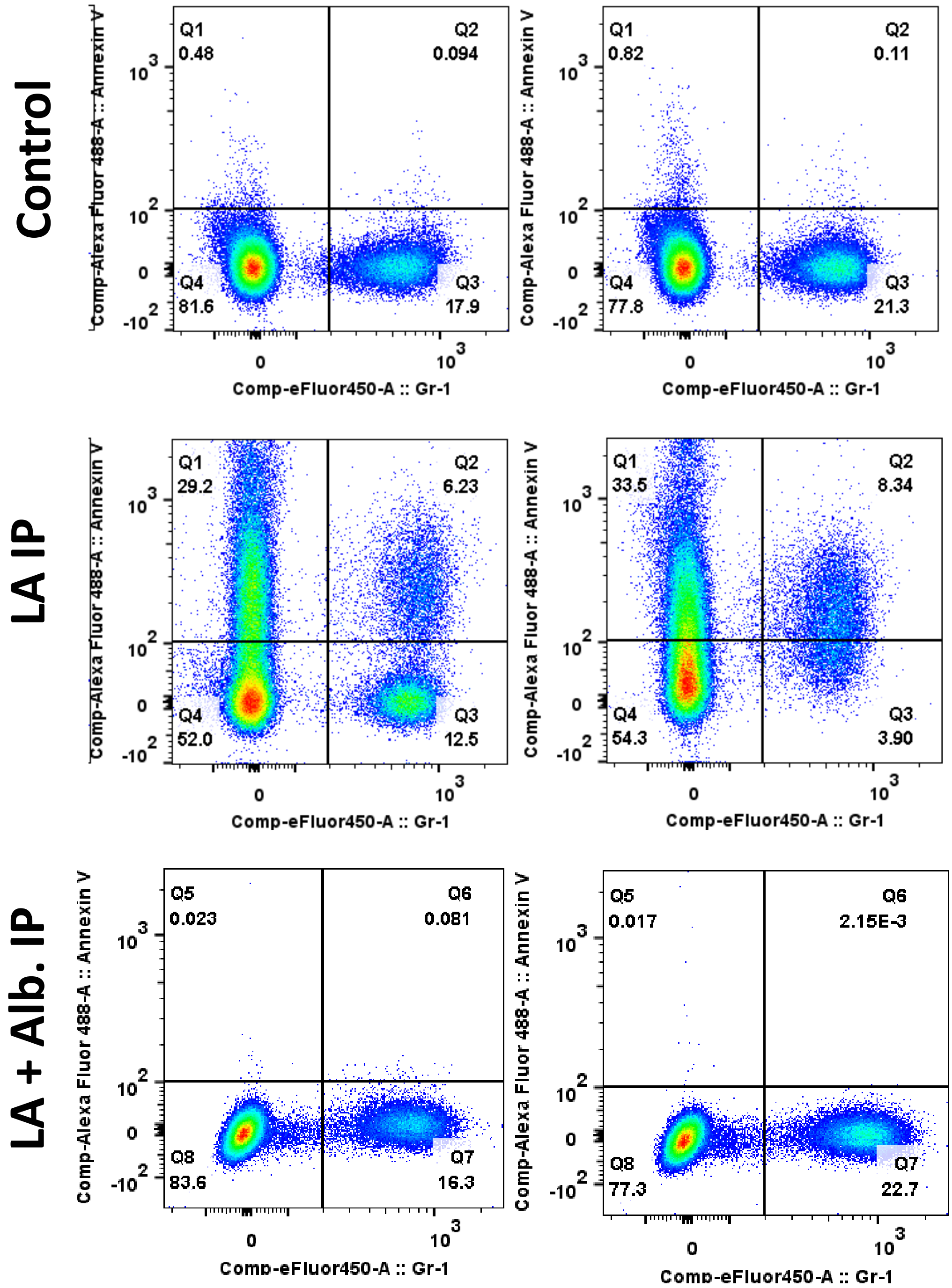

Supplemental figure 9

CD3 positive cells

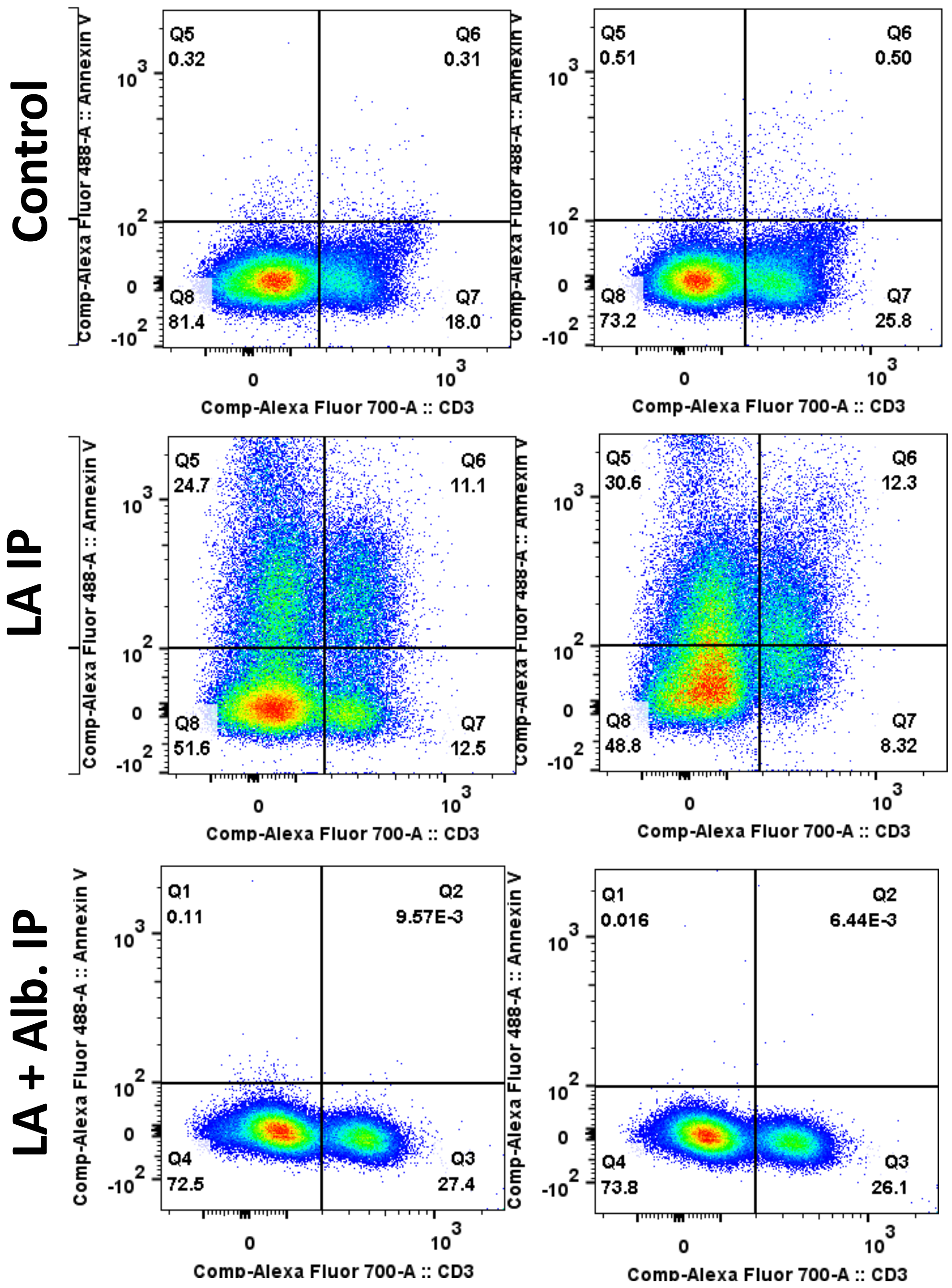

Supplementary figure 10

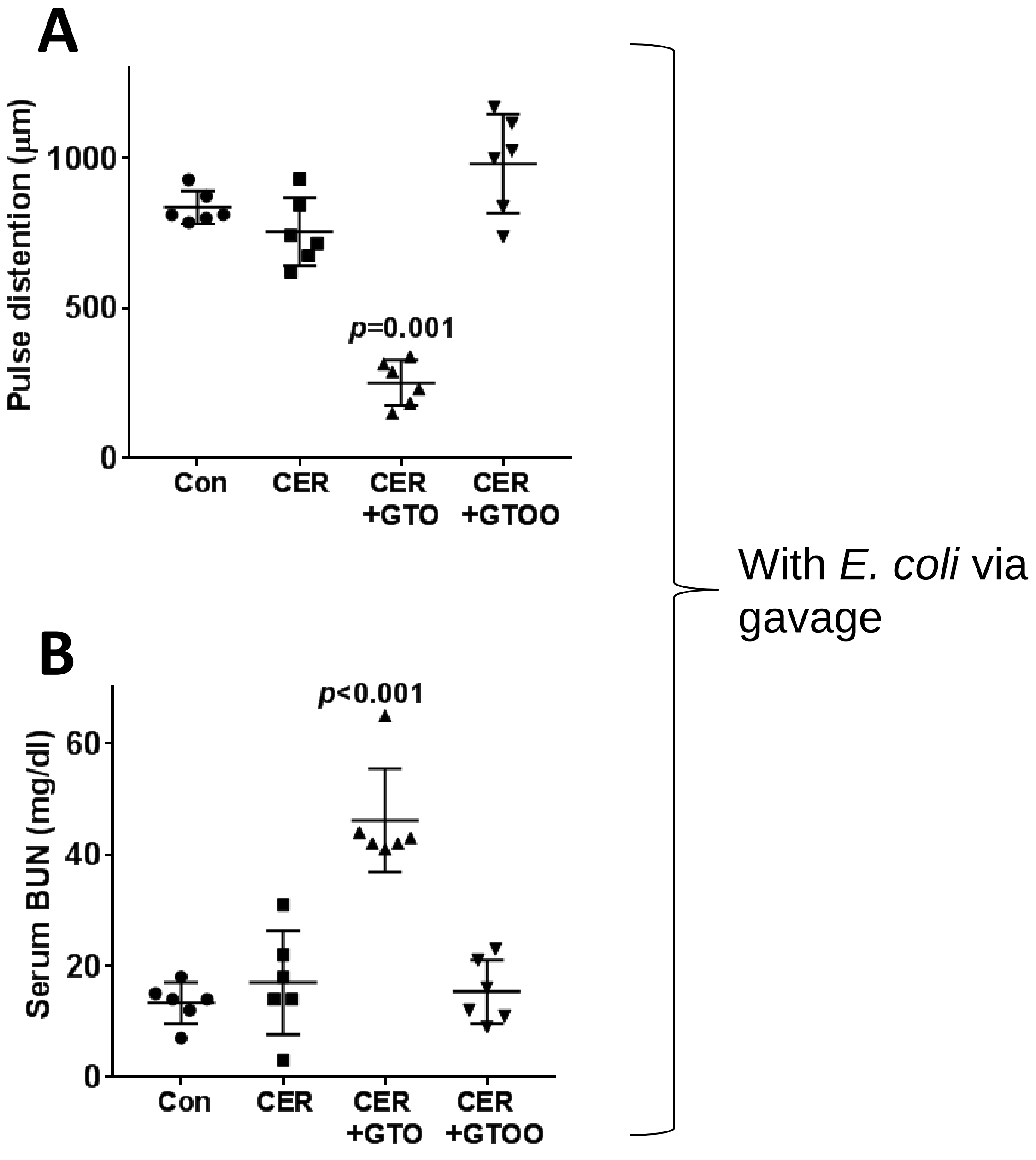

Supplemental figure 11

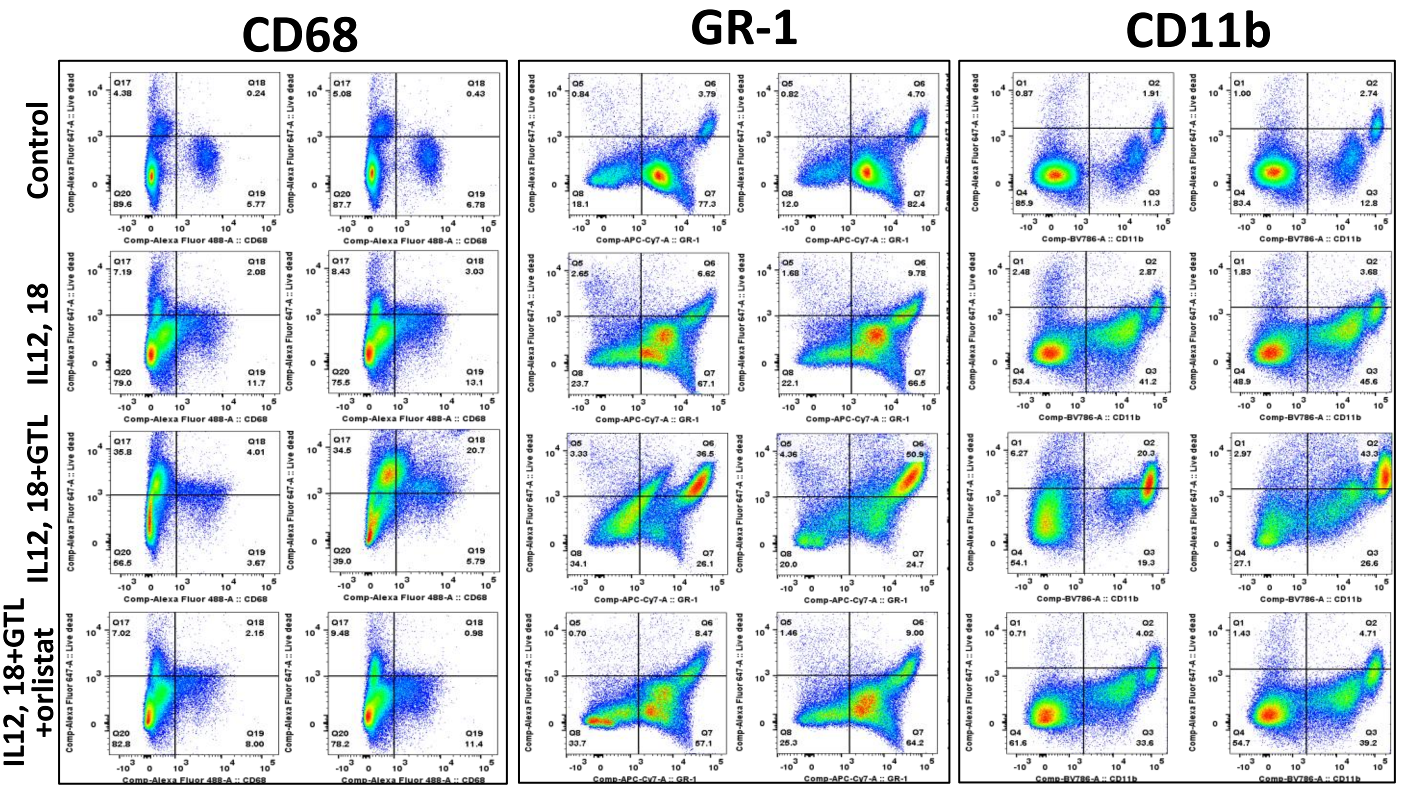

Supplemental figure 12

Histograms of live-dead marker +ve population

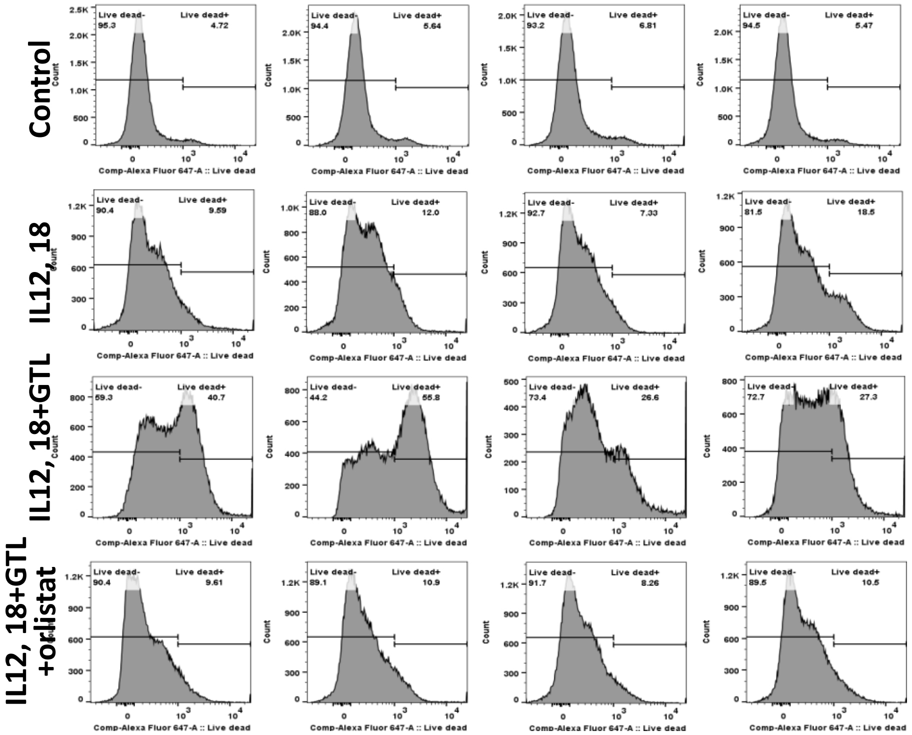

	Total Live dead positive
Control	4.7
Control	5.6
Control	6.8
Control	5.5
IL12&18 only	9.6
IL12&18 only	12.0
IL12&18 only	7.3
IL12&18 only	18.5
IL12&18 +GTL	40.7
IL12&18 +GTL	55.8
IL12&18 +GTL	26.6
IL12&18 +GTL	27.6
IL12&18 +GTL + Orli	9.6
IL12&18 +GTL + Orli	10.9
IL12&18 +GTL + Orli	8.3
IL12&18 +GTL + Orli	10.5

Supplemental figure 13

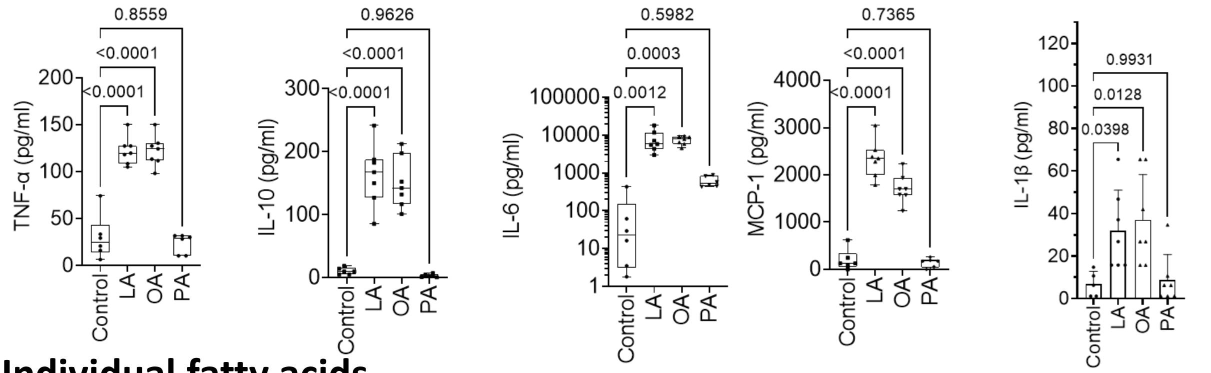

Individual fatty acids

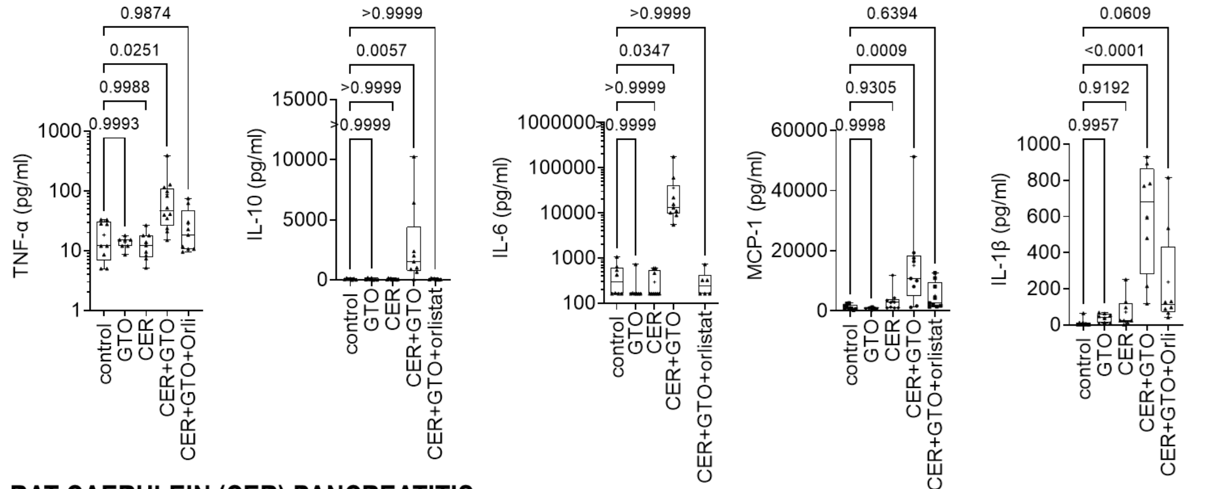

RAT CAERULEIN (CER) PANCREATITIS

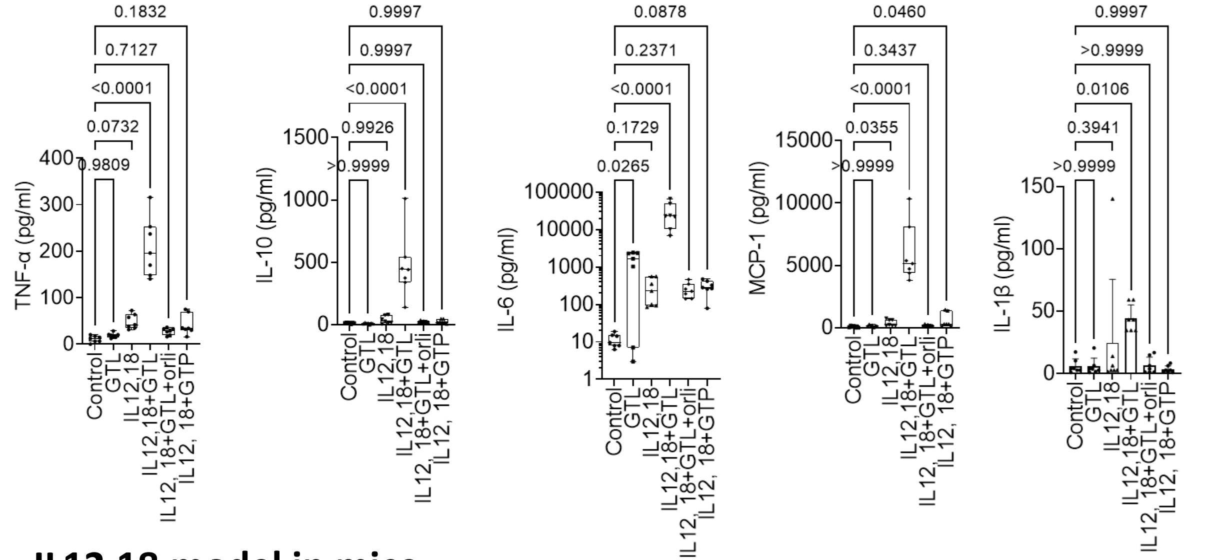

IL12,18 model in mice
